# Esophageal, gastric and colorectal cancers: Looking beyond classical serological biomarkers towards glycoproteomics-assisted precision oncology

**DOI:** 10.7150/thno.42480

**Published:** 2020-03-31

**Authors:** Elisabete Fernandes, Janine Sores, Sofia Cotton, Andreia Peixoto, Dylan Ferreira, Rui Freitas, Celso A. Reis, Lúcio Lara Santos, José Alexandre Ferreira

**Affiliations:** 1 Experimental Pathology and Therapeutics Group, Portuguese Institute of Oncology, 4200-162 Porto, Portugal;; 2 Institute of Biomedical Sciences Abel Salazar (ICBAS), University of Porto, 4050-013 Porto, Portugal;; 3 Institute for Research and Innovation in Health (i3s) , University of Porto, 4200-135 Porto, Portugal;; 4 Institute for Biomedical Engineering (INEB), Porto, Portugal, 4200-135 Porto, Portugal;; 5 Digestive Cancer Research Group, 1495-161 Algés, Portugal;; 6 Porto Comprehensive Cancer Centre (P.ccc), 4200-162 Porto, Portugal;; 7 QOPNA/LAQV, Departamento de Química da Universidade de Aveiro, Aveiro, Portugal;; 8 Department of Surgical Oncology, Portuguese Institute of Oncology of Porto, 4200-162 Porto, Portugal;; 9 Institute of Molecular Pathology and Immunology of the University of Porto (IPATIMUP), Porto, Portugal;; 10 Faculty of Medicine, University of Porto, Porto, Portugal.

**Keywords:** glycobiomarkers, glycoproteomics, glycomics, glycosylation, digestive tract cancer, precision oncology

## Abstract

Esophageal (OC), gastric (GC) and colorectal (CRC) cancers are amongst the digestive track tumors with higher incidence and mortality due to significant molecular heterogeneity. This constitutes a major challenge for patients' management at different levels, including non-invasive detection of the disease, prognostication, therapy selection, patient's follow-up and the introduction of improved and safer therapeutics. Nevertheless, important milestones have been accomplished pursuing the goal of molecular-based precision oncology. Over the past five years, high-throughput technologies have been used to interrogate tumors of distinct clinicopathological natures, generating large-scale biological datasets (*e.g.* genomics, transcriptomics, and proteomics). As a result, GC and CRC molecular subtypes have been established to assist patient stratification in the clinical settings. However, such molecular panels still require refinement and are yet to provide targetable biomarkers. In parallel, outstanding advances have been made regarding targeted therapeutics and immunotherapy, paving the way for improved patient care; nevertheless, important milestones towards treatment personalization and reduced off-target effects are also to be accomplished. Exploiting the cancer glycoproteome for unique molecular fingerprints generated by dramatic alterations in protein glycosylation may provide the necessary molecular rationale towards this end. Therefore, this review presents functional and clinical evidences supporting a reinvestigation of classical serological glycan biomarkers such as sialyl-Tn (STn) and sialyl-Lewis A (SLe^A^) antigens from a tumor glycoproteomics perspective. We anticipate that these glycobiomarkers that have so far been employed in non-invasive cancer prognostication may hold unexplored value for patients' management in precision oncology settings.

## Introduction

Esophageal (OC), gastric (GC) and colorectal (CRC) cancers are part of the top ten most prevalent and deadliest tumors worldwide 1. Despite representing a diversified array of diseases with distinct aetiologies and molecular backgrounds, these tumors share comparable management problems 2.

**Figure 1:**
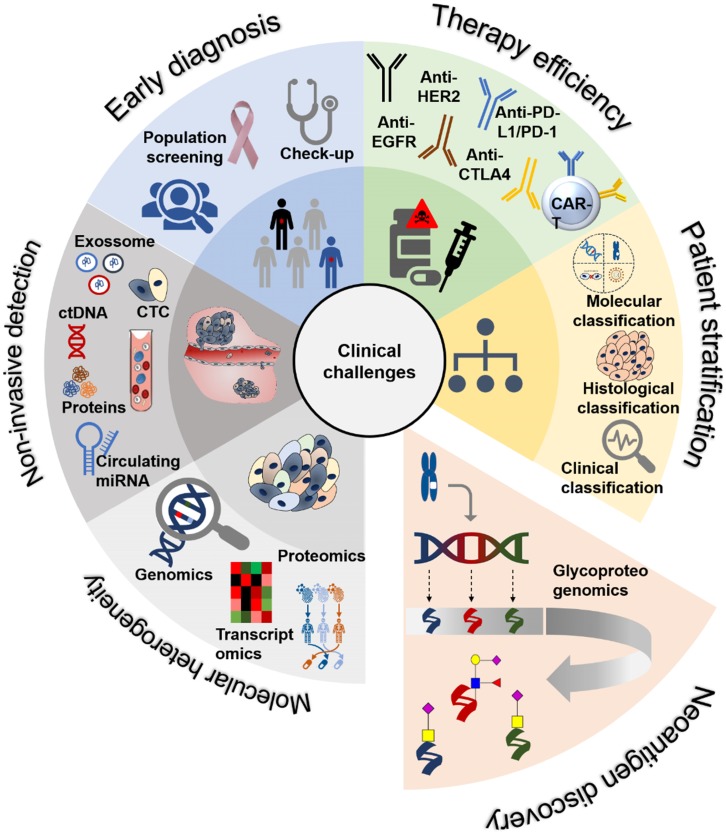
** Gastroesophageal and colorectal cancers main clinical challenges (inner circle), current clinical approaches (outer circle) and opportunities provided by neoantigen discovery.** Current clinical challenges include**: i) Lack of robust early diagnosis tools,** requiring population screening and timely medical check-ups, especially for population subsets presenting known risk factors (poor diet; *H. pylori* infection; family history, age, gender, pre-neoplastic lesions; etc). The absence of molecular biomarkers with the necessary specificity and sensitivity to assist in this matter remains a tremendous obstacle for early cancer detection; **ii) Need for patient stratification,** mostly achieved based on the clinicopathological classification of the lesions and, most recently, moving towards the incorporation of molecular biomarkers; **iii) Therapy selection and efficacy,** currently based on clinicopathological features but rapidly evolving towards molecular-assisted settings capable of aiding therapy personalization and early definition of responders. Therapeutic management has been based on surgery, chemo and/or radiotherapy, encompassing severe toxicity and limited efficacy, particularly for advanced disease stages. However, this paradigm has started to change with the introduction of antibody-based targeted therapeutics against key oncogenic cell surface receptors and immune-check point proteins such as PD-1, PD-L1 and CTLA4. CAR-T immunotherapy is also amongst future promising approaches**; iv) Non-invasive detection,** necessary for real-time monitoring of disease status and evolution throughout the course of disease. The field of liquid biopsies has tremendously evolved with the evaluation of circulating tumor DNA/miRNAs, proteins, micro and nanovesicles (exosomes and others) and, more recently, the study of circulating tumor cells (CTCs). The evaluation of these biomarkers in bodily fluids has improved prognostications and helped refining therapeutic selection, evaluating responses, establishing the risk of metastasis development and the detection of radiologically occult micrometastasis; **v) Molecular heterogeneity** is also a critical clinical challenge. This aspect has been a major obstacle towards effective molecular-assisted oncology and the introduction of targeted therapeutics. Nevertheless, the field has experienced significant advances with next generation sequencing, which generated a significant amount of genomics and transcriptomics data that has been used to propose gastric and colorectal cancer molecular subtypes. Cancer proteomics characterization has also contributed to the identification of relevant biomarkers; however, with yet limited clinical translation; **vi) Cancer neoantigens discovery** also represents a critical objective and a daunting challenge. It will be crucial for the identification of cancer-specific fingerprints capable of guiding therapeutic decision and designing effective targeted therapies and immunotherapy with very limited off-targeted effects. The comprehensive integration of genomics, transcriptomics and proteomics as well as information on post-translational modifications, with emphasis on glycosylation, will be of key importance for the identification of relevant protein functional nodes and targetable biomarkers at the cell-surface.

These include poor prognosis due to late diagnosis and the lack of efficient therapeutics to avoid disease progression, dissemination and metastasis. Early detection based on large-scale screening of symptomatic and asymptomatic populations remains a complex task, relying almost exclusively on upper digestive tract endoscopy and colonoscopy. The absence of reliable non-invasive molecular tools for cancer detection has significantly delayed the generalization of life-saving interventions to a wider population (**Figure 1**). Moreover, patient management is further aggravated by the molecular heterogeneity presented by gastroesophageal and colorectal tumors, even between lesions of apparently similar histological nature 3-5 (**Figure 1**). Such aspects impede accurate patient stratification and lead to ineffective therapeutic schemes that may be accompanied by severe toxicity, ultimately delaying or impairing more effective interventions. The identification of patients at higher risk of developing metastasis has also been particularly challenging, requiring a more profound knowledge of the complex molecular networks established by cancer cells and the tumor stroma (**Figure 1**). In fact, these aspects are critical for inducing cell migration and invasion, supporting the formation of pre-metastatic niches and circulating tumor cells that dictate metastatic spread 6. Another key challenge is therapeutic management. For most OC, GC and CRC lesions, first line therapy remains surgery and chemotherapy/radiotherapy 7. However, many subpopulations of cancer cells are endowed with chemotherapy resistance, leading to tumor relapse and unfavorable outcomes 8. The introduction of antibody-based targeted therapeutics with trastuzumab (anti-HER2), ramucirumab (anti-VEGFR2), and cetuximab (anti-EGFR) have increased patient survival in some subgroups, as highlighted by recent reviews 9. However, antibody-based targeted therapeutics efficiency may be considered modest, requiring refinements in patient selection and improvements in antibody- ligand recognition. The recent introduction of immune check-point inhibitors targeting PD-1/PD-L1 and CTL4 has constituted an exciting milestone in the field of immunotherapy, with yet conflicting results regarding patient's survival 10. Again, the existence of robust molecular models to predict patients better served by these approaches remains to be established, hindering true conclusions about therapeutic efficacy (**Figure 1**). It is also consensual that the multifactorial and dynamic molecular nature of gastroesophageal and colorectal tumors might benefit from combination therapies supported by real-time assessment of response and rapid adaptation of therapeutic schemes, which is yet to be accomplished.

The comprehensive interrogation of gastroesophageal and colorectal tumors using genomics and transcriptomics has already translated into predictive molecular models for GC and CRC, which will decisively shape future clinical practice towards precision oncology 11 (**Figure 1**). Notably, OC has been a neglected neoplasia regarding these objectives. In addition, genomics is being used to guide proteomics studies in GC and CRC envisaging targetable biomarkers and therapeutic personalization 12, 13. This constitutes the foundations for oncoproteogenomics that couples mass spectrometry (MS) approaches with high-throughput next-generation sequencing (NGS) to study the role of protein variants in biological processes and pathology, while identifying cancer neoantigens for therapy development. This big push for systems biology is expanding fast, being complemented with novel layers of molecular information translated by post-translational modifications (PTM), which are critical for biological systems regulation. While much research focus has been set on the role of phosphorylation, functional glycoproteomics has now demonstrated that glycosylation is also critical for defining key oncogenic features such as cell motility, invasion, metastasis and immune escape 14. Alterations in protein glycosylation also decisively contribute to the activation of relevant oncogenic pathways that sustain cell survival and proliferation 14. Moreover, cancer associated changes in the composition, density and distribution of glycosites are responsible for unique molecular signatures at the cell-surface holding tremendous potential for targeted therapeutics against membrane glycoproteins 15.

Two of the most studied glycans in OC, GC and CRC are the sialyl-Tn (STn) antigen, a short-chain glycan resulting from a premature stop in protein *O*-glycosylation 16, and the sialyl-Lewis A (sLe^A^) antigen 17, a terminal glycoepitope of carbohydrate chains in glycoproteins and glycolipids. These glycoepitopes are absent or very strictly expressed in healthy tissues, being significantly overexpressed in advanced stage digestive tract tumors where they are frequently associated with poor prognosis 18. STn and SLe^A^ antigens are widely explored in clinical practice for non-invasive follow-up based on the CA72-4 (for STn) and CA19-9 (for SLe^A^) tests; however, they lack the necessary specificity and sensitivity for diagnosis and accurate patient stratification 18. Nevertheless, there is a relevant amount of information supporting their involvement in cell invasion and metastasis development 19, providing a relevant rationale for intervention 15. Moreover, several pre-clinical and clinical studies involving targeted therapeutics and immunotherapy focused on these glycans show exciting results but also outstanding challenges towards clinical implementation. As such, herein we provide a comprehensive review on the functional implications and current clinical significance of STn and SLe^A^ in gastroesophageal and colorectal cancer. We also highlight the potential of revisiting these glycans from a glycoproteomics perspective and attempt to set critical research milestones for its integration in emerging panomics studies envisaging future exploitation in the context of precision medicine.

## Protein Glycosylation in Cancer: Structural Diversity and Biological Significance Facing Clinical Applications

The extracellular domains of most membrane- anchored proteins are glycosylated, often at multiple sites, creating a dense network of carbohydrate chains at the cell surface termed glycocalyx. There is growing evidence that the nature of cell surface glycosylation decisively impacts on the structure and function of membrane proteins and consequently on cell homeostasis 20. The cancer glycoproteome varies at different levels, including alterations in the density and distribution of glycosites and/or glycan chains structure 15. The most common glycosylation alterations in cancer, including in digestive tract tumors, comprise: i) changes in the substitution patterns of fucose and sialic acid residues; ii) dramatic reduction in *O*-GalNAc glycans extension (PTM of Ser and Thr residues) through oversialylation; and iii) alterations in branching, core fucosylation and sialylation of *N*-glycans (PTM of Asn); and iv) alterations in terminal glycan chain epitopes, including Lewis blood group patterns 21. Aberrant extracellular glycans may act as signal transducers, regulating key intracellular pathways involved in cell proliferation and survival 14, 22. Furthermore, glycosylation modifications may significantly impair the normal function of key proteins involved in cell-cell and cell-extracellular matrix adhesion such as integrins, cadherins and CD44 towards more motile and invasive phenotypes 15, 20, 23, 24. The glycocode also governs recognition by immune cell lectins, dictating immune response often towards more cancer tolerogenic phenotypes 25. Even though the molecular and clinical contexts driving changes in glycosylation remains, in many cases, unclear, it is quasi unanimous that the tumor microenvironment promotes glycophenotypes that favor disease progression and dissemination 14. Accordingly, the tumor microenvironment-tumor cell crosstalk is rapidly translated by cell surface glycosylation changes to drive stress adaptation or active escape 14, 26. Overall, glycans integrate extra- and intracellular cues that are phenotypically reflected at the cell surface, being active players in malignancy or surrogate markers of cellular adaptation. As such, glycans are attractive targets for theragnostic applications, which will be comprehensively addressed later in this review. Focus will be set on the most widely studied and clinically explored glycans in gastroesophageal and colorectal tumors, STn) and SLe^A^ antigens.

**Figure 2:**
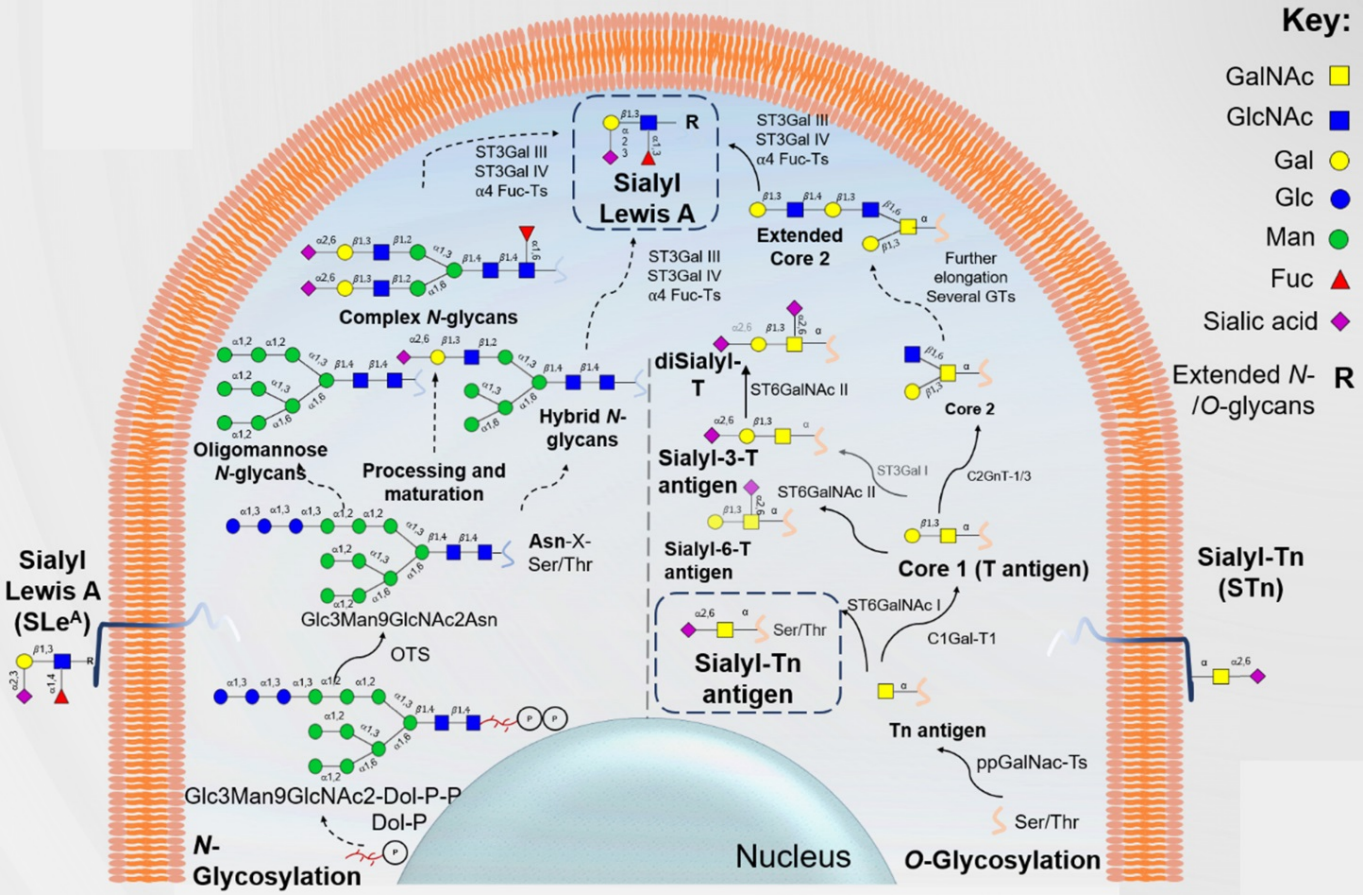
** Protein-associated glycan biosynthetic pathways.** *N*-glycan biosynthesis begins at the endoplasmic reticulum (ER) membrane with the Glc3Man9GlcNAc2-P-P-Dol precursor transfer to Asn in Asn-X-Ser/Thr sequons of proteins by oligosaccharyltransferase (OST). Of note, “X” represents any amino acid except Pro. Subsequent *N*-glycan processing reactions take place in the ER from where glycoproteins exit to the Golgi apparatus carrying *N*-glycans with either eight or nine Man residues, termed oligomannose *N*-glycans. Further, biosynthesis of hybrid and complex *N*-glycans occurs in the medial-Golgi. Subsequent sugar additions diversify the repertoire of hybrid and complex *N*-glycans by elongation of branching GlcNAc residues, capping of elongated branches and *N*-glycan core sugar addition. Common terminal structures include sialyl lewis antigens, such as sialyl lewis A (SLe^A^) through the concerted action of beta-galactoside-alpha-2,3-sialyltransferase-III and IV (ST3Gal III and IV) as well as alpha-4-fucosyltransferases (α4-FucT). Mucin type *O*-glycan biosynthesis is initiated in the late ER or in Golgi compartments by the Polypeptide GalNAcT- mediated transfer of *N*-acetylgalactosamine (GalNAc) to Ser or Thr residues of proteins in a tissue- and cell-type-specific manner. This first biosynthetic steps yield the Tn antigen (GalNAcα1-Ser/Thr), the simplest form of mucin-type *O*-glycosylation. Tn antigen can be sialylated into the sialyl Tn (STn) antigen by *N*-acetylgalactosaminide alpha-2,6-sialyltransferase I (ST6GalNAc I), abrogating further chain extension, or it can be extended into the core 1 antigen (T antigen) by *N*-acetylgalactosamine 3-beta-galactosyltransferase 1 (C1Gal-T1). In turn, the core 1 structure can be capped with sialic acid residues through the action of *N*-acetylgalactosaminide alpha-2,6-sialyltransferase 2 (ST6GalNAc II) or ST3Gal I, giving rise to sialyl T and disialyl T antigens, again preventing further extension. On the other hand, T antigen can be extended into the core 2 glycan by core 2 beta-*1*,6-*N*-acetylglucosaminyltransferase-I or III (C2GnT-I, III). Extended *O*-glycan structures beyond the core 2 antigen can also display terminal structures similar to *N*-glycan such as SLe^A^.

Over- or *de novo*- expression of STn (NeuAcα2-6GalNAcα1-*O-*Ser/Thr) antigen results from significant alterations in protein *O*-GalNAc glycosylation impairing its full extension and maturation. *O*-GalNAc glycosylation is a tightly regulated tissue-dependent process in which the density and distribution of glycosites is dictated by the nature of UDP-GalNAc:polypeptide *N*-acetylgalactosaminyl transferases (ppGalNAc-Ts) present in the cell 27. It starts with the addition of GalNAc residues to serine (Ser) or threonine (Thr) moieties by one of twenty possible ppGalNAc-Ts, originating the Tn antigen 28. The GalNAc precursor may be further extended into different core glycans that can give rise to more complex structures terminated by fucosylation and sialylation 21. Therefore, it is not uncommon to observe *O*-GalNAc glycans with terminal sialylated lewis blood group determinants such as the SLe^A^ antigen, particularly in the malignant gastrointestinal and colorectal mucosa 29, 30. On the other hand, the Tn antigen can be sialylated by ST6GalNAc-I or -II to form STn, which stops further glycan elongation and largely reduces the *O*-glycans repertoire at the cell surface 31 (**Figure 2**). Known mechanisms underlying increased STn levels include overexpression of *ST6GALNAC1* sialyltransferase gene 32 and mutation 33 or hypermethylation 34 of *COSMC*, compromising COSMC, a major T synthase chaperone. This impairs T antigen formation while making Tn antigen available for sialylation by ST6GalNAc-I 35. Golgi-to-endoplasmic reticulum relocation of polypeptide *N*-acetylgalactosamine-transferases (GalNAc-Ts) also drives Tn accumulation in cancer cells 36, ultimately favoring STn biosynthesis (**Figure 2**). Both Tn and STn antigens have been classically associated to mucins due to their high glycosite density and are frequently termed simple-mucin type *O*-GalNAc glycans 37. Nevertheless, virtually all glycoproteins presenting extracellular motifs prone to be *O*-glycosylated may express these glycoepitopes 38. However, STn expression in healthy tissues is normally restricted to poorly represented secretory cells 16, 31. It is also not expressed by most blood cells, with the exception of some lymphocyte populations 39. Contrastingly, STn is expressed by more than 80% of human carcinomas and, in all cases, it associated with adverse outcomes and decreased overall survival of patients 31, making it a potentially low risk biomarker for targeted therapeutics.

Cancer glycoconjugates at the cell-surface also frequently overexpress sialylated lewis-type blood group antigens such as SLe^A^ (Siaα2,3Galβ1,3(Fucα1,4)GlcNAc) and its isomer SLe^X^ (Siaα2,3Galβ1,4(Fucα1,3) GlcNAc) as terminal epitopes of *O*-glycans, *N*-glycans, and glycolipids 40, 41. However, SLe^A^ has been more prominent in the context of serological assays and cancer research because, contrarily to SLe^X^, it is not expressed by blood cells. Sialyl Lewis antigens biosynthesis is a complex process involving the coordinated action of several glycosyltransferases, which might vary depending on the nature of the glycoconjugate carrying the antigen (**Figure 2**). Increased SLe^A/X^ levels in *O*-glycans have been mostly associated with C2GnT overexpression 42, while aberrant glycolipid SLe^A/X^ has been correlated with the activation of β1,3-GlcNAc transferase 43. The competition between the biosynthesis of type 1 (SLe^A^) and type 2 (SLe^X^) chains is tissue specific and is mostly governed by β-1,3 and β-1,4-galactosyltransferases basal levels 44. During malignant transformation, overexpression of sialyl lewis antigens also derives from neo-synthesis or incomplete synthesis of more complex lewis structures. Namely, non-malignant digestive tract epithelial cells predominantly express di-sialyl Lewis A (disLe^A^) antigens, presenting an additional *O*-6 linked sialic acid than SLe^A^ 45. In healthy epitheliums, this isoform interacts with inhibitory sialic acid-binding immunoglobulin-type lectins (Siglecs) in immune cells, contributing to maintain tissue homeostasis 46, 47. Upon neoplastic transformation, epigenetic silencing of *ST6GALNAC6* due to histone deacetylation and DNA methylation leads to the expression of the short isoform SLe^A^. This results in loss of normal cell-cell recognition between mucosal epithelial cells and lymphoid cells and gain of E-selectin binding capacity of cancer cells, ultimately favoring immune escape and metastasis 47. Similarly, non-malignant colon cells predominantly yield sialyl-6-sulfo lewis X, while cancer cells often overexpress SLe^X^ 48. Malignant transformation drives this shift through downregulation of sulfate transporters 49, sulfotransferases and PAPS synthases 50. Overall, oncogenesis promotes the expression of sialylated lewis antigens, which constitute the minimum requirement for selectin binding to their counter-receptors, stablishing the basis for hematogenous metastasis 44.

In summary, there is a substantial amount of evidences supporting a functional role for STn and sialylated Lewis antigens, with emphasis on SLe^A^, in cancer development. Such rationale associated with a restricted expression pattern in healthy tissues, makes of these glycans a good starting point for developing targeted therapeutics. However, a more precise knowledge about the nature of the glycoproteins carrying these PTM signatures is required envisaging the molecular context for personalized intervention.

### Biological significance of STn antigen expression in cancer

**Figure 3:**
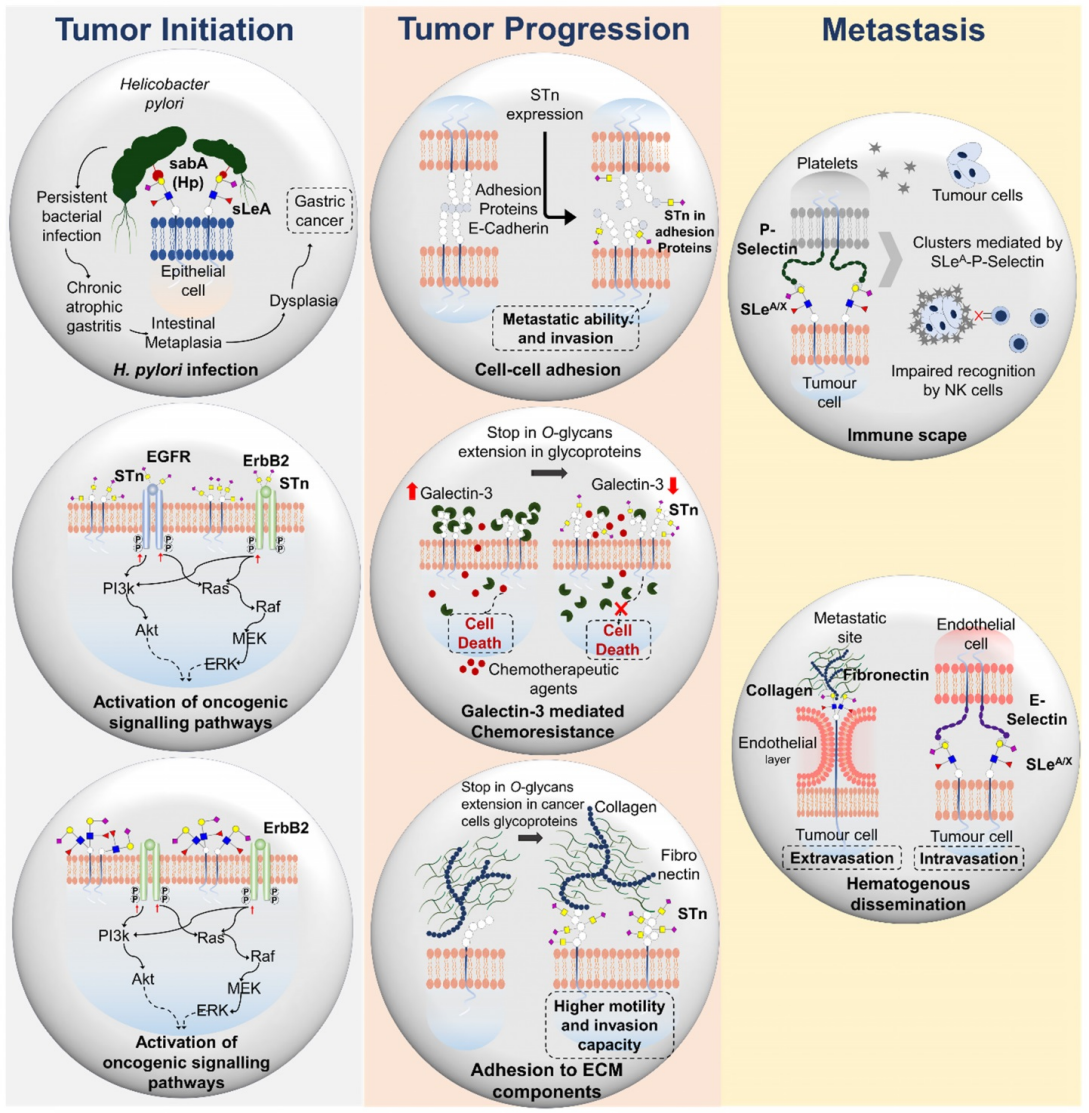
** Functional impact of STn and SLe^A^ expression in gastrointestinal tumors.** Both STn and SLe^A^ overexpression influence tumor initiating processes as constitutive activation of several oncogenic signalling mediate by EGFR and ErbB2. Moreover, SLe^A^ also facilitates *H. pylori* adhesion to the gastric epithelium, contributing to persistent infection and potentially cancer development. STn also drives tumor progression by negatively impacting cell-cell and cell-extracellular matrix adhesion as well as galectin-3-mediated chemoresistance. Moreover, sialylated lewis antigens facilitate hematogenous metastasis of tumor cells through E-selectin interactions, while protecting tumor cells from sheer stress in circulation and hampering immune recognition.

As previously described, aberrant modulation of glycosylation pathways in cancer cells can lead to the neo- and/or overexpression of STn antigen 31. STn expression has been described in many types of epithelial cancer, including OC 51, GC 52 and CRC 53, and a significant effort to disclose its functional role has been employed in the latest years. However, since cancer cells maintained *in vitro* rarely express the STn antigen, most functional studies have been done exploring cell models glycoengineered to expressed high levels of this glycan 19, 32, 54. Such observations emphasize the intricate relationship between STn expression and the tumor microenvironment 26, which remains to be fully disclosed. In fact, in bladder cancer, it has been suggested that decreased oxygen levels may promote STn biosynthesis by suppressing the expression of glycosyltransferases involved in *O*-glycans elongation 26; however, the microenvironmental factors driving STn expression should be further addressed in future studies. To this date, there are no functional studies on the role of STn in OC and most functional findings in CRC are inferred from *in situ* tissue studies. Nevertheless, GC cells glycoengineered to overexpress *ST6GALNAC1* and consequently the STn antigen, display enhanced invasion capacity *in vitro* and increased metastatic ability in nude mice than the corresponding negative controls 19, 55. Moreover, MUC1 and CD44 were found to be major STn carriers 19, 24, suggesting that STn might negatively impact on GC cell adhesion towards more metastatic phenotypes 55 (**Figure 3A**). Furthermore, GC cells induced to overexpress *ST6GALNAC1* and *ST6GALNAC2* displayed lower proliferation rates than control cells, mostly due to an increase in the percentage of apoptotic cells and not to a G1 cell cycle delay 19. STn expressing cells also showed markedly reduced homotypic cell-cell aggregation but increased adhesion to ECM components as collagen and fibronectin 19 (**Figure 3A**). Other studies have disclosed a novel STn-dependent mechanism for chemotherapeutic resistance in GC cells 56. Namely, STn increased galectin-3 intracellular accumulation by decreasing its interaction with cell surface glycan receptors. In this process, STn decreased chemotherapy sensitivity of cancer cells, highlighting the importance of developing novel strategies targeting galectin-3 and/or ST6GalNAc-I in GC 56 (**Figure 3A**). In addition, studies involving glycoengineered *COSMC* knock-outs demonstrated that *O*-glycan shortening was accompanied by the acquisition of mesenchymal-like traits, while promoting transcriptomic remodeling towards overexpression of *SRPX2* and *RUNX1* genes (both commonly upregulated in GC tissues) 54. STn overexpression also lead to the activation of ErbB tyrosine kinase receptors ErbB2 and EGFR in these models, implicating STn in the constitutive activation of oncogenic signaling in GC 54 (**Figure 3A**). *COSMC* knock-out mouse xenografts demonstrated decreased survival compared to wild type xenografts, vowing for the more aggressive nature of short-chain *O*-glycan overexpressing GC cells 54. These findings highlight the role of STn in the acquisition of malignant features related to increased migration, invasion and chemoresistance in GC. These findings suggest that the STn antigen may support metastasis development, a concept that is reinforced by its presence in circulating tumor cells 57 and metastases 58, 59 in different models.

In addition, overexpression of *ST6GALNAC1* enhanced STn expression in CRC stem cells (CSC) 60. Particularly, STn was carried by the CSC marker CD44, and increased the sphere-forming ability and resistance to chemotherapeutic agents of CRC-CSC 60. Furthermore, *ST6GANAC1*-overexpressing cells constitutively activated the Akt pathway, which was blocked by *LGALS3* (Galectin-3) gene knockdown. These findings suggest that *ST6GALNAC1* has a role in the maintenance of CRC-CSCs by activating the Akt pathway in cooperation with galectin-3 and that *ST6GALNAC1* or STn antigen might be reasonable molecular targets for CSC-targeted therapy 60. Similarly, other studies reported CD44 as a major STn carrier in colorectal cancer 61, suggesting that CD44 glycosylation may be implicated in cellular adhesion alterations. The observations made for GC and CRC agree with previous reports from different types of tumors (breast, ovarian, prostate, bladder) where STn was also implicated in key oncogenic features favoring invasion and metastasis development, supporting the pancarcinoma and aggressive nature of this PTM 15, 31, 62.

### Biological significance of SLe^A^ antigen expression in cancer

As previously mentioned, the overexpression of both SLe^A^ and SLe^X^ is associated with increased tumor cell malignancy. Particularly, these glycoepitopes are overexpressed in several types of cancer cells, including esophageal 63, gastric 64 and colorectal cells 65, 66, where its functional role is being progressively disclosed. Furthermore, SLe^A/X^ are E-selectin ligands in vascular endothelial cells, facilitating tumor cell intravasation into primary tumor blood vessels and circulating tumor cells arrest in distant locations during hematogenous metastasis 67, 68 (**Figure 3B**). Also, it potentiates tumor cell adhesive interactions with extracellular matrix proteins on vessel walls, mediating extravasation 69 (**Figure 3B**). Furthermore, interaction of these ligands in colon cancer cells with P-selectin on platelets is thought to facilitate hematogenous dissemination by protecting tumor cells from sheer stress in circulation and immune recognition by natural killer (NK) cells 70 (**Figure 3B**). Moreover, through this interaction, activated platelets may release platelet-derived growth factors capable of stimulating the growth of highly metastatic colon cancer cells 71. These observations suggest that SLe^A^ may play a relevant role in the survival of tumor cells in circulation, which warrants future confirmation. In addition, colon cancer cells undergoing epithelial-to-mesenchymal transition (EMT) upregulate SLe^A/X^ levels and E-selectin binding capacity, while enhancing VEGF production 72. These findings suggest a pro-angiogenic feedback resulting from cancer cells SLe^A/X^ and endothelial cells E-selectin interactions. The molecular mechanism underlying EMT-associated SLe^A/X^ expression in colon cancer cells included the transcriptomic regulation of glycogenes in a c-Myc and CDX2 dependent manner. Namely, the EMT-derived enhanced transcriptional activity of c-Myc resulted in upregulation of *ST3GAL1/3/4* and *FUT3*, while down- regulation of *CDX2* suppressed *FUT2* transcription, ultimately leading to SLe^A/X^ overexpression 72.

In GC cells, the ErbB2 tyrosine kinase receptor (RTK) was found to be a major carrier of SLe^A^, mostly in a *FUT3* dependent manner. Moreover, these cells displayed a hyperactivation of ErbB2 and other members of the ErbB family 20. However, whether ErbB2-specific glycosylation plays an active part on dimer formation remains to be fully disclosed, although similar associations have been reported for other ErbB receptors 73 (**Figure 3B**). Notwithstanding, cellular deglycosylation and CA19.9 antibody-mediated blocking of SLe^A^ drastically altered ErbB2 expression and activation, suggesting an important role for SLe^A^ in RTK function in GC cells 20. On another note, SLe^A/X^ are specific ligands of sialic acid binding adhesin (SabA) that promotes *H. pylori* adhesion to the inflamed gastric mucosa 29 (**Figure 3B**). Moreover, SLe^x^ expression in the gastric epithelium is further induced during persistent *H. pylori* infection, creating a chronic inflammation state capable of establishing precursor neoplastic lesions of the gastric epithelium 74. In this context, sialyl lewis antigens actively contribute to chronic inflammation and gastric carcinogenesis.

In summary, sialylated lewis antigens, with emphasis on SLe^A^, actively contribute to pre-malignant gastric lesions, constitutive activation of oncogenic pathways, hematogenous metastasis and immune scape. This supports the malignant nature of cancer-specific PTM and highlights the need for novel therapeutic approaches targeting these epitopes.

## Clinical Relevance of STn and SLe^A^ in Cancer

### Classical serological tests and non-invasive biopsies

**Figure 4:**
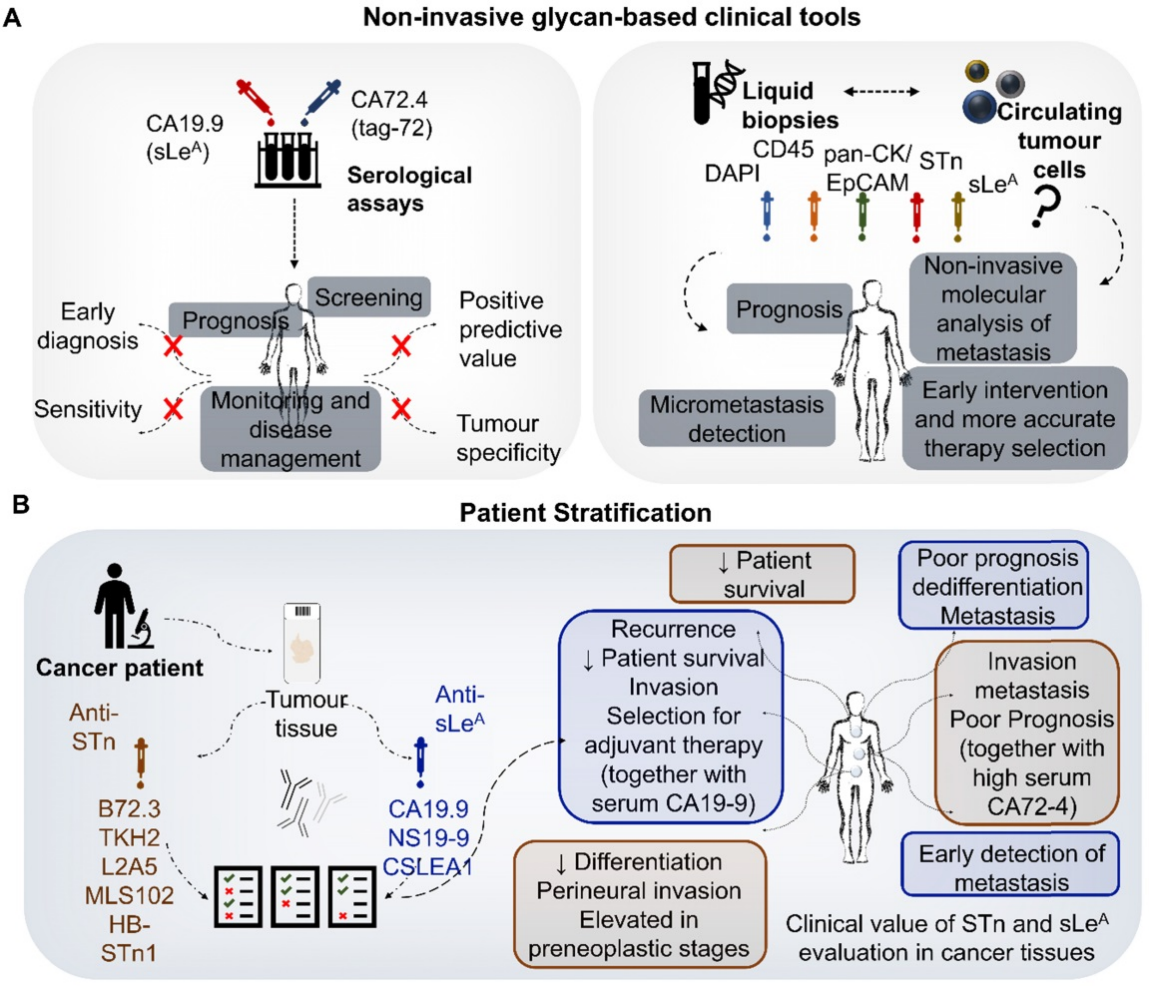
** STn and SLe^A^ clinical applications (non-invasive serological methods and molecular-assisted decisions using cancer tissues) in gastroesophageal and colorectal cancers. A) Non-invasive serological methods.** The STn antigen is detected in the serum using the CA72-4 test; whereas the SLe^A^ antigen can be detected by the CA19-9 test. These antigens are used for prognostication, cancer screening and response to therapy monitoring. However, both lack the sensitivity and specificity for early diagnosis; **B) Molecular-assisted oncology using cancer tissues.** The right side of B panel highlights some of the most explored monoclonal antibodies detecting STn (orange) and SLe^A^ (blue), while the left side summarizes known clinical associations.

Both STn and SLe^A^ antigens are often present in proteins that are secreted of shed from tumors into circulation, making them accessible for serological detection through the CA72-4 (for the STn antigen) and CA19-9 (for the SLe^A^ antigen) assays. These serological tests have been long introduced in clinical practice and frequently support the assessment of gastroesophageal and colorectal cancer patients' status (**Figure 4A**). The CA72-4 test detects a high molecular weight tumor-associated glycoprotein 72 (TAG-72) mucin carrying the STn antigen 75, whereas CA19-9 mainly detects mucins carrying SLe^A^ 76. However, in gastrointestinal tumor tissues, anti-CA19-9 antibodies may detect monosialogangliosides containing SLe^A^ 76, demonstrating possible cross reactions with a wide variety of SLe^A^-expressing glycoconjugates.

Recent metanalyses clearly associate CA72-4 and CA19-9 serum elevations in GC patients with higher stage and grade, vessel-invasion, lymph-node metastasis and distant metastasis 77. Despite showing potential for accessing recurrence, predicting patient survival, poor response to chemotherapy and monitoring after surgery 78 these markers are not considered suitable for early GC detection. In fact, both test show positivity related to non-malignant conditions, which decisively bias results. Namely, the CA72-4 antigen has been found elevated in the serum of approximately 7% of healthy individuals as well as patients with gastric ulcer, polyps, atrophic gastritis and *Helicobacter pylori* infection in a large study involving 7757 adults 79. These results indicate that routine screening of CA72-4 levels in asymptomatic patients may be ineffective due to low sensitivity and low positive predictive value 79. In addition, CA19-9 is synthesized and secreted in low amounts by normal human pancreatic and biliary ductal cells and by the gastric, colon, endometrial and salivary epithelia 80. It is also overexpressed in several benign gastrointestinal disorders, increasing the number of false positives 81. False negatives may also occur for individuals with Lewis (a-b-) genotype 80, which impacts negatively on the capacity to accurately discriminate cancer patients. Attempts to combine several classical serological biomarkers such as CEA, CA19-9 and CA72-4 in GC also did not provided satisfactory results for early diagnosis 79.

A large metanalysis involving 6434 CRC patients showed that high serum CA19-9 levels before treatment were significantly associated with decreased overall-, disease-, progression- and recurrence-free survival, irrespectively of the geographical region, analysis type, sample size, and treatment methods 82. Several studies also suggested similar associations for CA72-4, but the statistical power of these observations is still limited by the low number of studies 83. Nevertheless, the sensitivity of both biomarkers is also considered low to support its addition to current standard surveillance strategies for CRC 84. Contrasting with GC and CRC, the relevance of the CA72-4 and CA19-9 tests in OC is still poorly understood; nevertheless, the few existing studies also suggested associations with more aggressive disease status 85, 86. Moreover, high CA72-4 levels may be helpful to predict lymph node invasion in resettable esophagogastric junction adenocarcinomas 80. In summary, current data supports that CA72-4 and CA19-9 serum elevation is a molecular feature shared by subgroups of OC, GC and CRC patients presenting worst prognosis. Such observations denote a common background shared by tumors of distinct molecular and pathological natures. Nevertheless, there is still little information on the nature and origin of the proteins in circulation carrying these PTM, which will be critical to fully disclose and potentiate their clinical meaning.

Recently there has been a growing interest in exploiting circulating tumor cells (CTC) for prognostication, detection of micrometastasis and as a mean to obtain more accurate insights on the molecular nature of the metastasis, as emphasized by several recent reviews 87 (**Figure 4A**). Moreover, these cells have been observed in the blood of cancer patients with no evident radiological signs of metastasis 88, reinforcing their importance for early and potentially life-saving interventions. On a more comprehensive scale, CTC analysis may be used to complement circulating nucleic acid and exosome analysis 89, 90. Classically, CTC isolated from patients' blood by different means (flow cytometry; lab-on-a-chips) have been identified based on the expression of the epithelial markers EpCAM and pan-cytokeratins allied to the absence of CD45 hematopoietic marker 87, 91. Recently, we have demonstrated that the majority of CTC of different origins, including CRC express the STn antigen at the cell-surface and that targeting this glycan could greatly expand the number of isolated CTC, thus increasing the sensitivity of current CTC detection methods 57 (**Figure 4A**). Moreover, STn-CTC was identified in non-metastatic patients that subsequently experienced disease progression, further reinforcing the sensitivity of the methodology 57. The presence of STn in CTC also provided the missing link between STn promotion of motility, invasion and immune escape and its presence in distant metastasis of advanced tumors 57. Taking also into account the functional role of SLe^A^ in E-selectin mediated metastasis 44, it is likely that this glycan may also be present in CTC, which warrants evaluation. These observations are crucial to further improve liquid biopsies and should now be extended to larger well characterized patient cohorts. It also provides the rationale for selectively targeting CTC in clinical settings and developing novel glycan-based therapeutics capable of controlling disseminated disease.

### Expression in tumor tissues and metastasis

In clear contrast with serological assays, the evaluation of STn and SLe^A^ in tumor tissues is not part of the clinical routine. Envisaging the basis for glycoproteomics studies, we have comprehensively interrogated the literature regarding the clinical context of STn and SLe^A^ expressions in gastroesophageal and colorectal cancer **Table 1**.

To our knowledge, only one study has addressed the expression of STn in OC, using the B72.3 antibody which detects clusters of STn bound to serine and may also cross react with Tn clusters 92, 93. The authors reported that strong STn expression (> 35% of the tumor area) was predictive of decreased patient survival 94. Notably, there are also evidences that STn may be mildly present in squamous cells of the healthy esophagus 95, limiting the scope of these preliminary observations. In opposition to OC, the expression of STn in GC is well documented. Accordingly, STn overexpression was correlated with peritoneal dissemination and tumor undifferentiation 96. Moreover, most studies have strongly correlated STn immunoreactivity with decreased overall survival, with STn being demonstrated as an independent prognostic factor in some study settings 97, 98. STn staining has also been correlated with venous and lymphatic invasion, depth of invasion, tumor stage, and lymph node metastasis 58, 98. In addition, strong positive tissue immunoreactivity was associated with an elevation of serological CA72-4 58, suggesting shedding of tumor proteins into circulation. While Victorzon *et al.* suggested that STn was a valuable tumor marker capable of discriminating early stage patients with poor prognosis, Terashima *et al.* observed that STn was an independent prognostic factor in advanced GC patients. Notably, Yamashita et al, found no clinicopathological significance for STn expression in GC 99. Despite these discrepancies, most likely associated with experimental protocols and different cut-off levels of positivity, most studies support that STn antigen is directly linked to GC aggressiveness. Even though significantly overexpressed in GC, STn has also been observed in pre-malignant gastric lesions 100 and in lower amounts in particular cellular subsets in the healthy stomach 97, 101.

Studies in CRC reinforce the cancer-associated nature of STn, which was not detected in tumor-adjacent normal colorectal mucosa, apart from goblet cells 102-106. Interestingly, in the colon, STn can be detected in colonocytes after de-acetylation by saponification 107, 108. Moreover, decreased sialic acid *O*-acetylation on mucins was a sensitive indicator of early malignant transformation 109. In CRC tumors, STn was distributed in the apical cytoplasm or at the cell membranes, with staining becoming more pronounced with increasing extent of invasion 102. It was strongly correlated to poor histological differentiation and perineural invasion 104. STn was also consistently found in adenomas 104, 106 and transitional tissue, where it was significantly correlated with tumor stage according to the TNM and AJCC classifications 103. Altogether, these findings suggest that STn overexpression occurs early in the carcinogenesis process and STn could constitute a useful marker of the preneoplastic stage of colorectal tissue. Moreover, the different staining localization of STn may assist in distinguishing the process of malignant transformation from a diagnostic standpoint. Notably, the presence and extension of STn in CRC did not correlate with high levels of the antigen in the serum given by the CA72-4 test 110. Collectively the expression of STn in gastroesophageal and colorectal tumors appears to be linked to higher aggressiveness and metastasis development, in agreement with its functional role. Moreover, it holds potential for patient prognostication (**Figure 4B**). Nevertheless, more detailed studies involving larger and well characterized patient cohorts are required, particularly for OC. STn evaluation in the context of response to different types of therapeutic schemes, including targeted therapeutics and emerging immunotherapies with immune-check point inhibitors, are also warranted. This, together with the inclusion of STn in emerging stratification models, will be critical to improve patient stratification (**Figure 4B**). On the other hand, the presence of STn in healthy tissues, despite limited, raises concerns related to off-target effects of targeted therapeutics. A comprehensive characterization of the STn-glycoproteome will be critical to identify cancer-specific molecular signatures. Its comparison with the serum glycoproteome will also be decisive for explaining the discrepancies between tumor and serum status at this level and improving the accuracy and scope of the CA72-4 test.

To date, only three studies were identified involving squamous cell carcinomas (SCC), the most common OC histological type, and SLe^A^ antigen expression. SLe^A^ was consensually observed at the cell membrane and in the cytoplasm of malignant cells but not in the surrounding stromal tissue or in normal epithelium, suggesting a cancer-specific nature. Oshiba *et al.* 111 did not found any correlation between SLe^A^ expression and clinicopathological variables, while Ikeda, *et al.* 112 and Makino *et al.* 113 correlated SLe^A^ with tumor dedifferentiation, poor prognosis as well as hematogenous and distant lymph node metastasis, respectively. Differences in the clinical history of enrolled patients, including exposure to different therapeutic schemes prior to molecular evaluation, and adoption of different antibodies for SLe^A^ evaluation may account for the observed differences. In GC, SLe^A^ was observed at the membrane and cytoplasm of tumor cells but also in the stromal areas between tumor cell clusters. Undifferentiated carcinomas showing stromal staining were characterized by increased depth of invasion and tumor size 114. Moreover, these gastric tumors were shown to preferably follow the peritoneal dissemination type of metastatic route rather than liver metastasis 96. Accordingly, the staining pattern of SLe^A^ helped predicting the prognosis of patients with undifferentiated type advanced GC, while serving as a useful marker for the early detection of metastasis. In CRC, SLe^A^ expression in the primary tumor and stroma significantly correlated with several clinicopathological variables. Namely, lymph node metastasis, disease recurrence, decreased survival, poor histological differentiation, depth of invasion, venous invasion, advanced Astler- Coller's stage (C2) and advanced post- TNM stage (IV) 115, 116. However, some studies failed to find clinicopathological significance for SLe^A^ in CRC 117, 118. In addition, SLe^A^ antigen was found highly expressed in goblet cells of non-pathologic colon tissues 119. Notwithstanding, most studies stablished SLe^A^ as a useful marker of tumor aggressiveness and prognosis in patients with advanced CRC. In addition, some studies provided a comprehensive view on SLe^A^ status by evaluating both tissue and serum levels in CRC patients. Accordingly, SLe^A^ detection in tumor tissue and serum identified patients at high risk of cancer recurrence and death and could be useful to select patients for adjuvant therapy 110, 120. However, SLe^A^ serum levels were low sensitive for cancer patients, partly because genotypically Lewis^a-b-^ individuals (about 5-10% of the general population) cannot synthesize the SLe^A^ antigen 121.

In summary, despite some conflicting results, most stemming from the use of different antibodies, the majority of studies highlighted the cancer-associated nature of STn and SLe^A^, relating the presence of these antigens in the tumor and stroma with more unfavorable outcomes. Moreover, in most studies, the percentage of positively immunoassayed tumor samples exceeded 50% regardless of the tumor model, making these glycoepitopes representative targets. STn expression in tumors was mostly associated with depth of invasion and decreased overall survival, while SLe^A^ expression was mainly associated with lymph-node metastasis and disease recurrence. These findings are easily correlated with the functional roles of both antigens, in which STn promotes more motile and invasive tumor cell phenotypes and SLe^A^ is an active mediator of E-selectin mediated metastasis. Notably, most studies were carried out in the Japanese population, with limited representation of other ethnic groups. Moreover, some authors have reported differences in the prognostic value of some molecular markers in colorectal adenocarcinomas regarding the ethnic origin of the patients 122, 123. Also, of all mentioned studies, only Nakayama, *et al.* has included advanced CRC regional lymph node metastasis in their clinical sampling, bringing a broader notion of glycan staining in disease progression 116. Moreover, all studies concerning these antigens have been performed more than fifteen years ago, requiring a comprehensive revision facing updated clinicopathological and molecular classifications. Future studies should also involve the analysis of broader patient cohorts, the standardization of protocols, the inclusion of samples representative from all stages of disease, and the adoption of antibodies with well-defined glycan recognition patterns. Namely, the existence of a wide number of different antibodies with distinct affinities for glycan moieties has constituted a major drawback for biomarker research. Namely, over the years, different antibodies have been developed against STn antigen, targeting either clustered or monomeric STn residues dependent or independently of the linkage to the peptide backbone, and frequently showing cross-reactivity with other short-chain *O*-glycans such as the Tn and *O*-6 sialoepitopes 92, 93, 107. Moreover, the nature of immunogens has been diverse, including membrane fractions of breast cancer metastasis (clone B72.3), LS-180 colonic cancer cells (clone MLS102) and ovine submaxillary mucins (clones TKH2; HB-STn1; L2A5). For SLe^A^ this is even more critical, since it is a terminal epitope of different glycan chains of distinct glycoconjugates (*O*-glycans and *N*-glycans in glycoproteins; glycolipids), widely diversifying the array of possible immunogens. A comprehensive understanding about the nature of the cancer-associated glycoproteins carrying these PTM is also required for the rational design of novel antibodies of biomedical and clinical utility.

## Glycan-based Therapeutic Opportunities

### Glycan-based immunotherapy

The cell-surface nature of glycans and glycoconjugates (glycoproteins and glycolipids) holds great potential for developing targeted therapeutics, including selective drug delivery, precise inhibition of key oncogenic pathways and immunotherapy 18, 21, 124.

**Figure 5:**
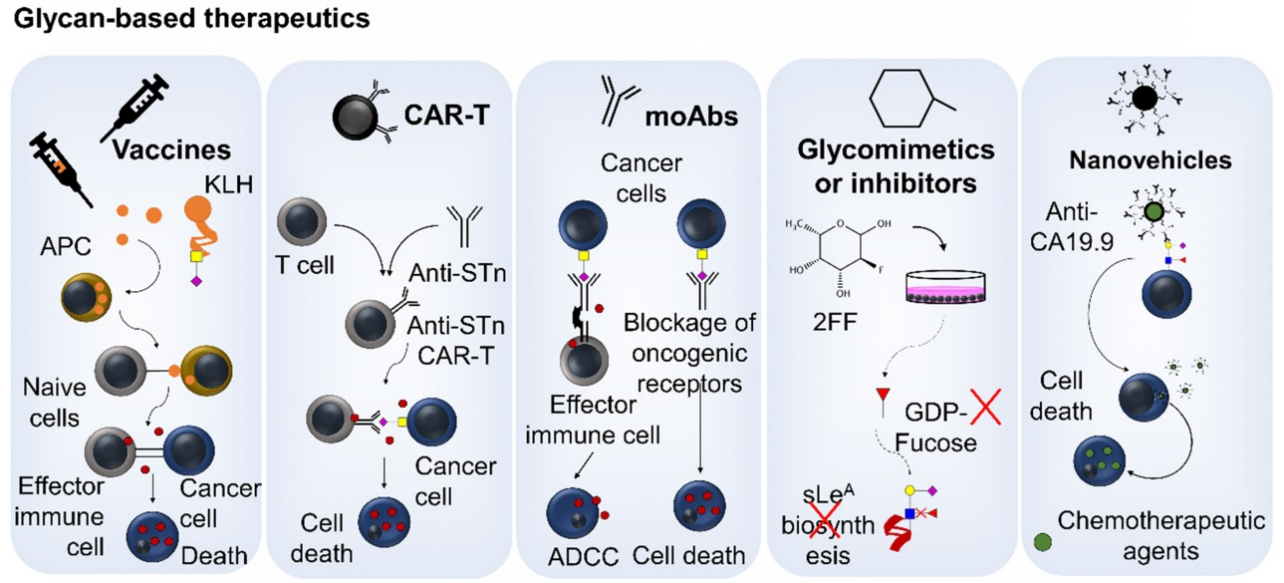
** Glycan-based therapeutics.** This includes glycan-based vaccines such as Theratope, exploiting the STn-antigen linked to a KLH protein carrier to elicited immune responses against STn-expressing cancer cells through antibody mediated killing and cytotoxic T cells effects. Other emerging immunotherapy is based on CAR-T cells engineered to target cancer cells expressing abnormal glycosylation. There are also several monoclonal antibodies capable of targeting abnormally glycosylated cells promoting antibody-dependent cellular cytotoxicity (ADCC) or blocking relevant oncogenic receptors at the cell-surface. Glycomimetics able to interfere with glycan biosynthesis or blocking glycan-receptor interactions relevant in cancer have also been developed. Finally, antibodies targeting glycans have been used to guide nanoparticles to tumor sites; thereby improving therapeutic outcomes.

Immunotherapy based on vaccination with short-chain cancer associated glycans is an appealing concept already explored in clinical trials, even though not for gastroesophageal and colorectal cancers (**Figure 5**). A pentavalent carbohydrate-based vaccine bearing several carbohydrate antigens, including STn, on a single polypeptide backbone, conjugated to keyhole limpet hemocyanin (KLH) and mixed with the QS-21 immunological adjuvant has entered in phase 1 clinical trial for patients with ovarian, fallopian tube and peritoneal cancers 125. However, the most promising approach continues to be Theratope, an STn-KLH vaccine that reached phase 3 clinical trials for metastatic breast cancer. Despite well tolerated by the patients, vaccination did not translate into an overall benefit in terms of time to progression and overall survival 126, 127. However, it has been suggested that a prior knowledge of the STn status of the tumors could have been crucial for patient selection and study outcome 128. The efficacy of these approaches could also be compromised by immunological barriers raised by the STn antigen. In fact, cancer-associated glycans as STn present variable immunogenicity depending on the distribution and nature of the glycopeptide chain 129. Moreover, STn may directly induce immune tolerance, including limited dendritic cell differentiation and induction of T-cell-mediated immunity, which are crucial for efficient cancer therapy 130, 131. As an example, densely glycosylated MUC1 sialoglycopeptides, frequently explored in the context of vaccine development, cannot be processed by antigen- presenting cells 132, impairing antigen presentation and consequent T cell activation. Nevertheless, these studies provide important lessons for choosing more adequate glycoepitopes, setting again the emphasis on glycoproteomics. There have also been attempts to overcome glycan-induced immune tolerance by coupling multiple carbohydrate antigens to specific carriers to form either clustered and/or multi-epitope conjugated vaccines 133. Glycans have also been combined with T-cell derived peptides or immunoadjuvant epitopes to produce glycoconjugate vaccines of multicomponent nature 134. Another strategy involves chemical modifications of glycans to improve immunogenicity. As an example, Song *et al.* recently investigated the antitumor ability of KLH-conjugated fluorinated STn analogues against a murine model of colon cancer 135. According to the authors, vaccine constructs with substitution of two *N*-acetyl by *N*-fluoroacetyl groups in STn significantly prolonged mice survival and reduced tumor burden in the lungs compared with Theratope (STn-KLH). The fluorinated vaccine elicited stronger cytotoxic T cell and Th1 immune responses and tumor-specific anti-STn antibodies capable of inducing complement and antibody-dependent cell-mediated cytotoxicity against human tumor cells, even in the absence of an immune adjuvant 135. Collectively, these findings suggest that strategic hapten fluorination may significantly improve the efficacy of glycan-based vaccines, even though the exact mechanism governing this immune response remains unknown. In addition, Ragupathi *et al.* described a SLe^A^ vaccine construct analogous to Theratope but containing a pentenyl glycoside of SLe^A^ hexasaccharide conjugated to KLH 136. This vaccine adjuvated by GPI-0100 induced high IgG and IgM titers responsible by mediating potent complement mediated cytotoxicity against different cancer cells. Moreover, the authors reported no cross-reactivity against other blood-group related antigens, including SLe^X^. In addition, MUC1-derived glycopeptides associated with cancer are amongst the array of glycoepitopes used in vaccines explored in clinical trials 137; however, with yet limited success. It was proposed that after first vaccination both tumor MUC1 and MHC molecules were reduced, suggesting an upfront response against these cells that was followed by therapy scape 138. These findings highlight the need to include a diversified array of glycopeptides that mirror tumor diversity. However, surrogate T-cell pre-activation outside the tumor bed, either in culture or by repetitive vaccination, could overcome tumor escape in MUC1 transgenic mice, offering an alternative approach to improve therapeutic schemes. Furthermore, the effective development of MUC1-based vaccines would be of great interest for patients with gastroesophageal and colorectal tumors that significantly overexpress abnormal glycoforms of this protein 139, 140. Collectively these studies demonstrate the feasibility of glycan-based anti-cancer vaccines but also highlight the importance of more accurate epitope choice and improved vehicle design, which requires a more profound knowledge of glycan-immune system interactions. The introduction of distinct T helper cell epitopes, Toll-like receptor agonists and other relevant immunogens in vaccine constructs together with the use of liposomes and nanoparticles as delivery systems may also help paving the way for improved vaccine designs 141, 142.

Another emerging approach relates with exploiting chimeric antigen receptor (CAR) T cells engineered to target glycosylated moieties in cancer cells, promoting selective cell death 143 (**Figure 5**). Despite boosted by advances in cell engineering, this is an old concept explored for therapeutic proposes in oncoglycobiology. In fact, the first-generation of CAR-T retrovirally transduced to efficiently target the TAG-72 glycoprotein in gastrointestinal tumor cell lines dates back to the nineties 144. Nevertheless, the concept has only been recently translated into a clinical trial for metastatic CRC 145. However, CAR-T cells were not able to elicit clinical response, potentially due to CAR antigenicity related to the murine origin of the scFv 146, lack of T cell co-stimulatory signaling, vowing for the inclusion of co-stimulatory molecules in CAR design, or the modest affinity of the CC49 anti-STn monoclonal antibody explored by this study 147. In addition, Loureiro *et al.* has recently reported that CAR-T cells can be efficiently and safely targeted to STn-expressing cells exploiting the recently developed L2A5 monoclonal antibody 148. These have been effective against breast- and bladder-associated tumor cells both *in vitro* and *in vivo*, but were not yet tested in digestive tract tumors 39. Loureiro *et al.* also reports some degree of cross-reactivity between the most explored anti-STn antibodies and immune cells. According to this study, L2A5, B72.3 and 3F1 (also referred to as HB-STn-1) showed no affinity for NK cells; however, B72.3 and 3F1 reacted with CD4+ and CD8+ T cells but not B cells. The L2A5 antibody recognized B cells and showed weak binding to a subpopulation of CD4low T cells 39. These critical observations demonstrated that CAR-T cells based on these antibodies carry the potential risk of fratricidal activities against T- and/or B lymphocytes. There are also concerns that STn-targeted CAR-T may significantly react against inflamed tissues, known to upregulate this glycan 149. Collectively, these observations suggest that, even though vaccination and consequent induction of circulating STn antibodies have been proven safe 150, more potent CAR-T therapy might encompass significant and potentially limiting off-target effects. Consequently, the future of glyco-targeted CAR-T remains dependent on the identification of targetable glyconeoantigens.

### Antibody-based therapeutics

Several monoclonal antibodies exist to target both STn and SLe^A^ antigens 151, 152, which may be used to induce antibody-dependent cellular cytotoxicity (ADCC) 153, 154, a mechanism by which many clinically available therapeutic antibodies promote anti-tumor effects 155, or block relevant oncogenic receptors 20 (**Figure 5**). These antibodies have been key tools for biomedical research but have shown limitations for theranostics (cancer detection and therapy), including guiding drugs, CAR-Ts and immunotherapeutic agents. Again, the refinement of the glycoimmunogens poses as a critical milestone towards this end. In addition, the identification of glycoepitopes involved in interactions with the immune system may lead to the development of novel antibodies for immune check-point inhibition; however, this remains a rather unexplored field of research. As such, antibody-targeted therapies for glycoconjugates remain intimately dependent on the development of bispecific antibodies targeting glycodomains in functionally relevant proteins.

### Glycosylation inhibitors and mimetics

Another appealing concept is to selectively inhibit glycan-receptor interactions or abrogate glycan biosynthesis pathways (**Figure 5**). As an example, the GMI-1271 E-selectin antagonist may be used to inhibit SLe^A/X^-expressing cancer cells adhesion to endothelial cells, consequently preventing metastasis development 156. This approach is currently being exploited for acute myeloid leukemia in phase III clinical trials (NCT03616470) 157; however, given marked similarities in metastasis routes, this approach may be translatable for solid tumors. Other phase 1/2 clinical trial (NCT02952989) was testing 2-fluorofucose (2-FF) 158, a known fucosylation inhibitor 159, together with pembrolizumab (anti-PD-1) in a wide array of tumors, including gastroesophageal junction adenocarcinomas, colorectal neoplasms and gastric adenocarcinoma. However, the trial was prematurely terminated due to overall benefit/risk profile. There are also promising attempts to use sialic acid mimetics to abrogate sialylation, interfering with sialylated-cell receptors that mediate immune-suppressive environments, ultimately enhancing cytotoxic T cell tumor immunity 160. These studies support the current relevance of glycomimetics in cancer treatment; however, concerns exist that their untargeted nature may significantly interfere with glycan-mediated cellular homeostasis, which warrants more in-depth evaluation. Nevertheless, conjugation of the molecules with monoclonal antibodies may provide the necessary means to target them to cancer cells.

### Glycan-targeted nanovehicles

Targeting nanovehicles carrying therapeutic molecules (chemotherapy, siRNA, small molecules of different natures, etc) to cancer cells is also an appealing therapeutic strategy (**Figure 5**); however, glycosylation has been less explored for this purpose. Nevertheless, we have recently developed a biocompatible targeted nanoparticle for selective delivery of chemotherapy (5-FU and paclitaxel) to SLe^A^-expressing GC cells, with minimal off-target affinity for healthy tissues. We anticipate that the preferential accumulation of nanomolecular constructs in tumor sites, due to ineffective vasculature and poor lymphatic drainage, associated to its targeted effect may significantly improve the controlled release of different agents in tumor sites with minimal toxicity for other organs 161. The refinement of these solutions *in vivo* and the adoption of bispecific monoclonal antibodies for targeting may pave the way to reduce the adverse systemic effects associated with chemotherapy, while enabling the administration of biologically effective drug dosages. These approaches may be particularly critical for elderly populations that generally do not tolerate conventional chemotherapy.

From another perspective, a prior knowledge about the structural alterations and functional implications of glycosylation in known cancer targets may help reshaping current antibody-based targeted therapeutics. In fact, many cell surface receptors such as HER2, VEGFR2 and EGFR explored in the clinics are heavily *O*- and/or *N*-glycosylated and may be prone to glycome alterations with the progression of disease 22. Ultimately, these glycoproteins may present STn or SLe^A^ antigens 20, 22, which warrants careful investigation. In addition, glycans influence PD-1/PD-L1 interactions 162. Namely, PD-L1 glycosylation stabilizes and modulates its binding to PD-1 163 and efforts are ongoing to exploit the glycoepitopes mediating these processes for patient stratification 164 A comprehensive glycomapping of these proteins may be helpful for early identification of treatment-responders and designing more appropriate antibodies.

## Glycoproteomics towards Precision Oncology

**Figure 6:**
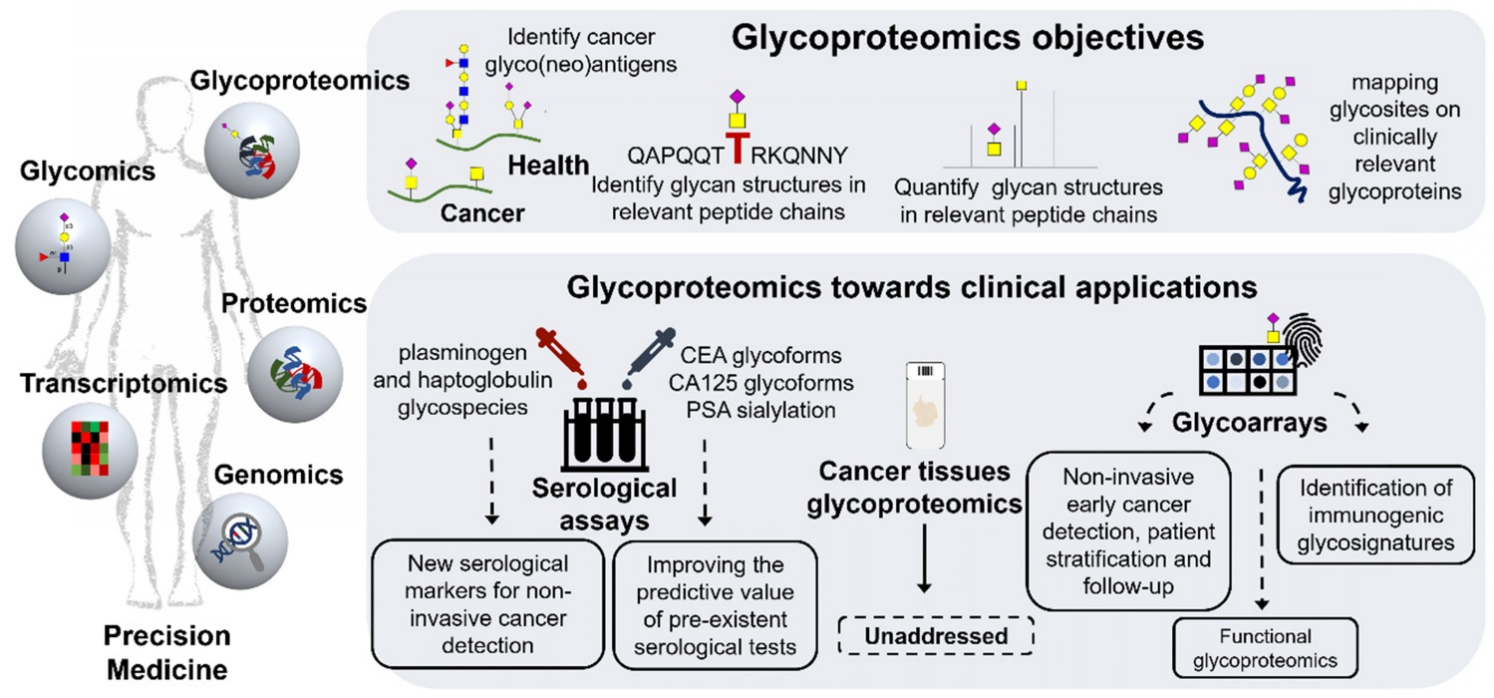
** Glycoproteomics objectives and opportunities facing clinical applications.** The right top panel summarizes the objectives of glycoproteomics. Glycoproteomics provides an opportunity for identifying cancer unique molecular fingerprints at the cell-surface (glyco”neo”antigens) that are not reflected by healthy cells and non-malignant conditions, paving the way for precise cancer targeting. Alterations in glycan composition, glycosites density and distribution associated to peptide domains in clinically relevant glycoproteins may significantly contribute to this end. The bottom panel highlights the main findings achieved by glycoproteomics in gastroesophageal and colorectal tumors to this date. It mostly includes serological studies, with emphasis on the identification of alterations in the glycosylation of plasminogen and haptoglobulin showing potential for non-invasive gastric and esophageal cancer detection and early diagnosis. Targeting specific CEA and CA125 glycoforms may also improve the predictive value of existing clinical tests. In addition, glycopeptide arrays bearing different types of protein glycoforms immobilized in solid supports have shown potential to improve early cancer detection and prognosis based on the identification of autoantibodies. It may also be a relevant tool for identifying potentially immunogenic protein glycoforms as well as a decisive device for functional assays. Contrastingly, cancer tissues glycoproteomics is yet to be initiated, which will be critical foreseeing true clinical applications.

The characterization of protein glycosylation is crucial for better understanding the complexity of biological systems and of the outmost importance for next-generation cancer biomarker discovery. In fact, several reports demonstrate that cancer cells present unique glycopeptide repertoires that may significantly improve the clinical value of classical biomarkers. Typical examples include targeting alterations in *N*-glycan antennas, fucosylation and sialylation in prostate specific antigen (PSA) to improve the PSA test limited sensitivity for non-invasive detection of prostate cancer 165-167 or exploring the glycosylation state of MUC16 (CA125) for ovarian cancer 168 (**Figure 6**). However, neither the genome, transcriptome nor the proteome can individually predict the structural nature, distribution and dynamics of glycan chains in proteins. Glycoproteomics attempts to bridge this gap by addressing the glycome,* i.e.* the repertoire of glycans in each biological milieu, as it appears in the proteome. This includes defining which glycosites on a glycoprotein are occupied, as well as identifying and, ideally, quantifying the glycan structures in a peptide chain (**Figure 6**). Such goal requires an adaptation of conventional proteomics protocols to accommodate the structural diversity of glycan chains and glycopeptides, which may be particularly challenging since the same protein may exhibit a myriad of different glycoforms. From an analytical perspective, the presence of glycans may significantly decrease the efficacy of proteolysis steps required for conventional protein identification by bottom-up proteomics 169. Moreover, it may negatively influence glycopeptides ionization with consequent reduction in sensitivity in most mass spectrometers 170. As such, addressing the glycoproteome may be at the same time a stimulating and daunting enterprise. However, recent analytical advances have significantly improved glycosites annotation capacity, providing an important tool for glycoproteome characterization 171. Nevertheless, a prior knowledge of the glycome is often required to guide glycoproteomics research, enabling the adoption of adequate glycoprotein enrichment methods and MS ionization strategies 172. In addition, it generates key structural information that tremendously facilitates the annotation of glycopeptides and glycosites using spectral information from tandem MS experiments 173. These technologies are now sufficiently mature, especially for glycomics, and backed by important protocol guidelines to support clinical studies 174.

For a long time, altered glycosylation in the gastroesophageal and colorectal tracts was mostly associated with mucins due to their high *O*-glycosite density and overexpression in cancer 175. These events were regarded as responsible for amplifying alterations occurring in glycosylation pathways, including the events leading to STn and SLe^A^ overexpression. However, recent developments in glycoengineering exploiting zinc finger nuclease targeting of *COSMC*, a C1GalT1 chaperone that controls *O*-glycan elongation, has generated several cell lines expressing the Tn and STn antigens, including GC models 54, 176. This enabled the identification of several hundreds of proteins and thousands of glycosites, which greatly expanded our understanding of GC cell *O*-glycoproteome 38, 177, 178. Moreover, it demonstrated that abnormal glycosylation such as Tn and STn may occur in hundreds of human proteins, including classical cancer biomarkers such as CD44 24. Downstream studies on gastric and colorectal tumors set the rationale supporting the decisive role of these glycans in protein function favoring cancer progression and dissemination (acquisition of mesenchymal traits, activation of oncogenic pathways, higher metastatic potential) 14, 179, 180. Surprisingly, the glycoproteome of gastroesophageal and colorectal tumors remains unexplored and most of the studies involving these models are of serological nature with the goal of improving non-invasive cancer detection. Namely, Gomes *et al.* has used targeted glycoproteomics focusing on short-chain T and STn antigens to study a subset of patients with gastritis, incomplete and complete intestinal metaplasia and healthy individuals, envisaging biomarkers of GC precursor lesions 100. The STn antigen was found in circulating plasminogen in patients with gastritis and incomplete metaplasia, mimicking the expression of STn in gastric tissues 100. Notably, plasminogen has been associated with chronic infection of the gastric mucosa by *H. pylori* and plays a role in extracellular matrix remodeling and degradation, suggesting that altered plasminogen glycosylation in the serum may be the reflection of pre-malignant alterations occurring in the gastric microenvironment 100, 181. This study also clearly emphasizes that revising classical glycan biomarkers from a glycoproteomics perspective may generate a wide array of molecular signatures holding potential for improving non-invasive monitoring of populations at higher risk of developing GC. In addition, Kim *et al.* demonstrated that, haptoglobulin isolated from the serum of GC patients presented unique glycopeptide fingerprints in comparison to healthy controls, including specific alterations in the fucosylation of *N*-glycan antennas 182. Notably, many of the observed cancer-associated glycans were sialylated Lewis epitopes that could contribute to CA19-9 elevation; nevertheless, this aspect has not been fully clarified. Haptoglobulin is one of the most important and abundant acute-phase serological proteins 183 and alterations in its glycosylation patterns have been previously reported for an onset of different types of inflammatory diseases and cancers, including CRC 184. Explorative OC glycoproteomics also involving serum samples has pointed out in the same direction. According to Mann *et al.*, cancer induces several alterations in the abundance of glycoproteins involved with local and systemic inflammatory responses, such as haptoglobulin, α-1-acid glycoprotein, fetuin B and proteins associated with extracellular matrix remodeling such as collagen subunits, EMILIN-2, amongst others 185, 186. These studies also reveal glycopeptide signatures presenting changes in *N-*glycosylation related with overfucosylation and sialylation associated with OC and precursor lesions 185, 186. For CRC we emphasize an explorative study using lectin-enrichment that highlights elevations in sialylation and fucosylation in complement C3, histidine-rich glycoprotein, and kininogen-1 in comparison to adenoma and normal tissues 187. Finally, there have been attempts to focus on specific circulating cancer-associated glycoproteins such as the carcinoembryonic antigen (CEA), produced in gastrointestinal tissues during fetal development, lost after birth and recapitulated in cancer 188. The CEA serological assay has been used in clinical practice to monitor response to therapy and recurrence after surgery in CRC 189 and in pre-operative settings to aid tumor staging, treatment definition and prognosis 190. Nevertheless, it presents limited sensitivity facing early stage disease detection 191 and is falsely elevated in the first weeks after chemotherapy 192. Moreover, it is elevated in non-cancer-related conditions, limiting its application to population screening 193. However, glycomics studies demonstrate the existence of tumor-specific CEA glycoforms that may be exploited to improve its predictive potential 194. Nevertheless, collectively, serological studies conducted to this date demonstrate that the serum glycome and glycoproteome may be primarily shaped by glycoproteins resulting from systemic inflammatory responses and, to less extent, by proteins originating from tumor cells and stroma 195, 196. This fact may help explaining the lack of sensitivity of most common glycan-based tests and the importance of zooming in on the glycoproteome for abnormally glycosylated proteins or even specific peptide domains associated to the disease status.

Exploiting a different perspective, serological studies have shown the existence of humoral responses against abnormally glycosylated cancer-associated proteins in CRC patients. Pedersen *et al.* used mucin glycopeptide arrays to demonstrate the existence of autoantibodies against aberrant glycopeptides derived from MUC1 and MUC5 with Tn, STn and core 3 glycans in the serum of cancer patients. The authors describe that the cumulative sensitivity of the array analysis was 79% with a specificity of 92% for CRC detection 129. Moreover, autoantibodies to truncated Core3-MUC1 could be detected in both CRC and inflammatory bowel disease, suggesting this may constitute early steps of altered glycosylation in colon tissues that are not reflected in the healthy condition. In addition, our group has used patients' autoantibodies to demonstrate the existence of protein signatures carrying the SLe^A^ antigen in OC that changed according to the severity of the disease; however, the nature of these glycoproteins remains to be elucidated 197. These preliminary observations strongly suggest that glycosylation may contribute to originate cancer neoantigens that ultimately lead to an immune response; however, effective tumor elimination by the immune system is likely not to occur due to immunosuppressive cues promoted by short-chain *O*-glycans. Nevertheless, the notion prevails that humoral response may be used to pinpoint cancer-associated glycan signatures.

In summary, the glycoproteome of gastroesophageal and CRC tissues remains unaddressed. The studies conducted so far are focused on glycoengineered cell lines or patient's serum and are of explorative analytical nature. Moreover, most included a very low number of patients of diversified clinical histories and employed distinct protocols. Nevertheless, there are strong suggestions that cancer induces profound alterations in the serum glycoproteome that are not reflected in healthy and benign conditions. Notably, most of the identified alterations relate with systemic inflammatory responses and extracellular matrix remodeling accompanying cancer development, rather than specific proteins originated from tumor cells. A refinement of these studies by targeted approaches for cancer-associated glycans may provide the necessary settings for improving further on the potential of glycan-based non-invasive detection of digestive tract tumors. It is also surprising that the significant functional and clinical rationale involving STn and SLe^A^ antigens has not yet been translated in a targeted interrogation of gastroesophageal and colorectal glycoproteomes, including primary lesions as well as the metastasis. This has significantly delayed a more comprehensive understanding of cancer progression and impacts negatively on the capacity to develop bi-specific targeted interventions, exploiting the cancer-specific nature translated by bridging the glycome and the proteome.

## Panomics for Cancer Biomarkers Discovery: Where Do Glycoproteomics Fit Facing Precision Oncology?

**Figure 7:**
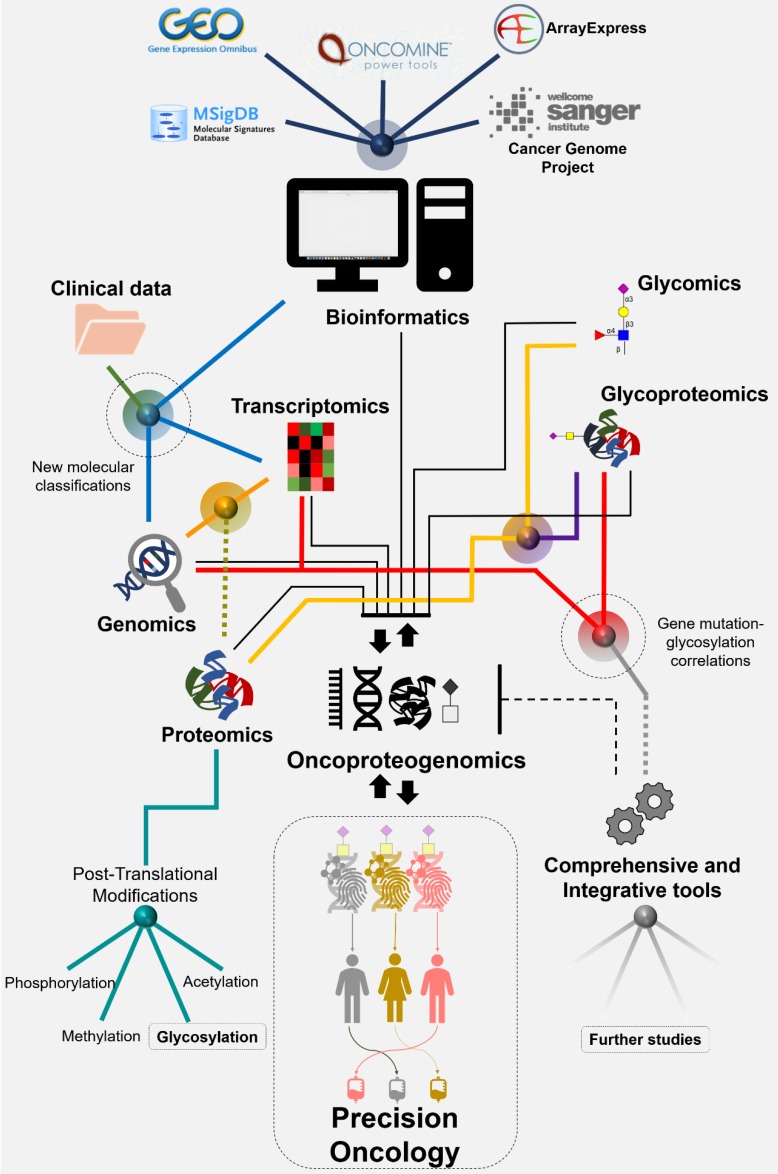
** The quest for molecular-assisted precision oncology can be found in the crossroad between interdependent omics (genomics, transcriptomics and proteomics with PTM analysis) backed by comprehensive bioinformatics settings.** This panel shows the possible connections between different omics, attempting to emphasize the importance of bringing together genomics, transcriptomics and proteomics allied to PTM (phosphorylation, methylation, acetylation and glycosylation, amongst others) analysis. It also aims to highlight the decisive role played by bioinformatics and current omics databases, which paved the way for tailored oncoproteogenomics. Namely, the comprehensive integration of genomics intel in customised databases can now greatly expand the coverage of protein annotations envisaging cancer neoantigens. This comprehensive strategy would be of key importance for accurate tumor stratification as well as identification of functional protein nodes and neoantigens for precise cancer detection and therapeutic design.

Biomarker research has experienced tremendous evolution over the past five years to accommodate the requirements of precision oncology. As a result, classical strategies focusing on a limited number of targets are being progressively replaced by systems biology frameworks propelled by high-throughput omics technologies. The technological readiness of next-generation sequencing platforms (genomics, transcriptomics) associated to consolidated bioinformatics and improved computational capacity has enabled the screening of large patient cohorts (**Figure 7**). Genomics and transcriptomics data have been made available in either raw (primary databases) and/or curated formats (secondary databases) in web repositories such as Gene Expression Omnibus (GEO), ArrayExpress and Oncomine enabling fast access, data integration and reinterpretation. In addition, databases such as Molecular Signatures Database (MSigDB), Cancer Genome Project, among others, have been exclusively created to gather molecular information related with cancer. As a result, transcriptomics studies on gastric and CRC have been recently reinterpreted and served as bases to stablish consensus molecular subtypes (CMS) with distinct clinical behaviors 198. Namely, in CRC, the CMS1 subtype is immunogenic and hypermutated. CMS2 tumors are activated by the WNT-β-catenin pathway and have the highest overall survival. CMS3 feature a metabolic cancer phenotype and CMS4 cancers have the worst survival, while having a strong stromal gene signature 198. In turn, the Asian Cancer Research Group (ACRG) reported four molecular subtypes of GC with clinical significance based on mRNA expression profiles: microsatellite-stable (MSS)/TP53-, MSS/TP53+, MSI, and epithelial-to- mesenchymal transition (EMT) subtypes. In this molecular classification, the MSI subtype was consistently associated with favorable prognosis, while EMT GC showed significantly higher recurrence rate, higher probability of peritoneal seeding at recurrence, younger age at presentation, and poorer survival compared to other subtypes 199. Notwithstanding, the predictive value of these models is being substantially refined through the continuous integration of clinicopathological data.

Despite the promising nature of multiomics approaches, the spotlight has been mostly centered on the genome and transcriptome, which often fail to fully reflect the proteome. A common example relates with reported discrepancies between gene expression and protein abundance at different levels 200. On the other hand, cancer proteomics is still constrained in its capacity to identify the products of single- nucleotide variants, fusion genes and alternative splicing, amongst other relevant events that alter protein primary sequences. Such limitations are associated with the lack of representation of genomic alterations in the databases conventionally used for protein annotation from tandem (MS/MS) mass spectrometry experiments. This limits the capacity of identifying cancer-specific signatures at the protein level, including neoantigens that hold potential for targeted therapeutics. In recent years, oncoproteomics initiatives are attempting to bridge the genome and the proteome using customized protein databases inferred from genomics (**Figure 7**). The conceptual bases of the approach are beyond the scope of this review but can be found comprehensively detailed in recent publications 201, 202. Notably, GC and CRC oncoproteogenomics has revealed the vulnerabilities and improved on currently established molecular models, while allowing prioritizing several targets previously inferred by genomic analysis, including copy-number drivers and mutation-derived neoantigens. Collectively, oncoproteogenomics decisively demonstrated that the combination of different levels of molecular information is of key importance for targeted intervention.

While oncoproteogenomics sets itself as the next cornerstone in cancer biomarker research, it becomes clear that including post-translational modifications (PTM) in comprehensive panomics models is crucial for better understanding biological milieus. PTM are not direct gene products but they exponentially expand the molecular and functional diversity of the proteome. PTM also facilitate rapid biologic adjustments to altered physiologic demands by enabling rapid incorporation of microenvironmental cues into protein function. For example, phosphorylation status regulates the activation of protein functions and acetylation and methylation of histones play a key role in epigenetic regulation of gene transcription, amongst other factors. Showcasing PTM importance, Vasaikar *et al.* recently identified Rb phosphorylation as a key oncogenic driver of proliferation and decreased apoptosis, providing the rationale for intervention in CRC 13. Very recently, Dong-Gi Mun *et al.* provided a proteogenomic analysis of diffuse GC in young populations by integrating mRNA, protein, phosphorylation, and *N*-glycosylation data 203. Interestingly, clear gene mutation-glycosylation correlations have emerged. Namely, in samples containing significantly mutated *TP53*, *CDH1*, *ARID1A*, and *RHOA, N*-glycosylation levels were significantly increased. Moreover, the proteins with such increased *N*-glycosylation were significantly associated with cell migration and immune response-related processes. Similarly, somatic mutations of genes involved in focal and integrin-mediated adhesion, regulation of actin cytoskeleton, FGF, PI3K-AKT, integrin, and Toll-like receptor signaling, as well as ECM-receptor and integrin interactions were associated with the *N*-glycosylation of proteins involved in the same processes 203. These findings suggest that *N*-glycosylation data, like phosphorylation data, provide additional information regarding associations of mutations with cancer-related cellular processes, reinforcing the key importance of integrating PTM in comprehensive molecular models. Overall, this study demonstrated that the distinction of tumor subtypes would not have been attainable through transcriptomics alone, again reinforcing the need for combining distinct molecular data. This report is also pioneer in terms of including glycosylation into comprehensive multiomics models contemplating multiple sources of molecular information (genome, transcriptome, (phospho)proteome). These observations also support the importance of continuing to pursue the analysis of the glycome and glycoproteome together with other molecular features in future studies. Nevertheless, the inclusion of glycoproteomics, with emphasis on STn- and SLe^A^ targeted studies, in comprehensive panomics models remains an open field of research of unquestionable potential.

## Concluding Remarks and Future Perspectives

**Figure 8:**
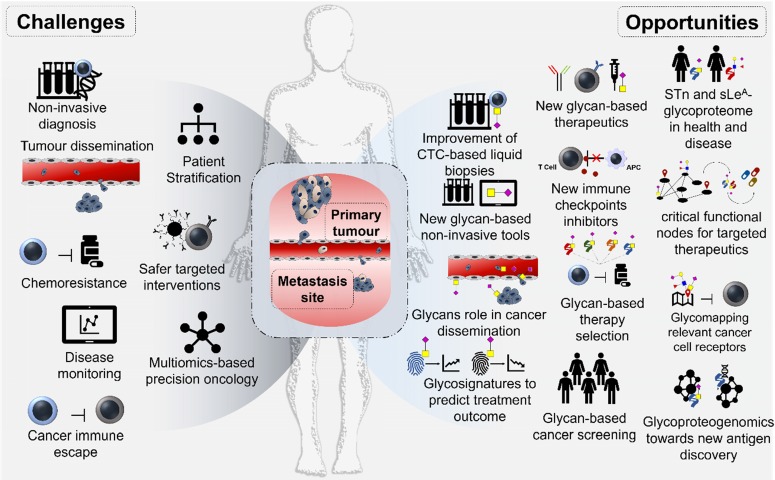
** Main cancer challenges tackled by targeted glycoproteomics and glycan-based opportunities facing clinical translation**. Glycans, particularly the STn and SLeA antigens, have been explored in the context of non-invasive cancer diagnosis, patient stratification, response to chemotherapy, disease monitoring, understanding and addressing immune response to cancer cells and design of safer targeted therapeutics. These approaches have been challenged by the lack of tumor specificity of these glycans; nevertheless, these glycobiomarkers specificity is being refined based on the integration of multiomics approaches. Fulfilling this objective will pave the way for improvements in liquid biopsies, targeted therapeutics, non-invasive cancer detection tools, patient stratification and prognostication models, novel targeted therapeutics, new immune check-point inhibitors, cancer glyconeoantigens and ultimately an improved understanding on the role of glycans in health and disease.

Gastroesophageal and CRC patients share similar therapeutic challenges that may be significantly attenuated by a better understanding of their molecular nature. Systems biology approaches bringing together different omics with clinical data are considered the next cornerstone for tumor molecular subtyping, enabling the identification of critical protein functional nodes for targeted intervention. Over the past five years, important milestones have been achieved towards this end, including elegant demonstrations that accurate patient stratification can only be achieved through the incorporation of multiple layers of biomolecular information (genome, transcriptome, proteome, PTM). In particular, the development of potentially curable actions is strongly dependent on precise patient stratification and the identification of neoantigens suitable for targeted approaches with minimal off-targeted effects. This is a challenging analytical task to perform at the protein level, requiring genomics-customized proteomics workflows and the incorporation of PTM, which are critical vectors for defining protein function in response to microenvironmental cues (**Figure 8**). Exploring alterations in protein glycosylation occurring at the cell-surface constitute the next logical step towards this end. Over the past twenty years a significant amount of functional and clinical data has been generated linking STn and SLe^A^ antigens to cancer aggressiveness in many distinct models, including gastroesophageal and colorectal carcinomas. As a result, serological tests have been created for these antigens and, despite sensitivity and specificity limitations, they are still explored in clinical practice for prognosis and patient monitoring. Very recently, STn was discovered in CTC and preliminary studies suggested it could be useful for improving the sensitivity of this type of liquid biopsies, while paving the way for targeted interventions. The analysis of solid tumors points in the same direction, strongly reinforcing associations between these glycans and poor outcome. Despite the scarce number of reports involving OC, STn and SLe^A^ antigens appear to present a pancarcinoma nature, suggesting common microenvironmental drivers that are yet to be disclosed. In parallel, functional studies involving different types of cell lines demonstrated that the replacement of extended *O*-glycans by short sialylated glycosides, as STn, or changes in terminal glycan epitopes, as sialylated Lewis determinants, decisively influences metastasis. Namely, this occurs by glycan-promoted enhanced motility, invasion of the extracellular matrix and neighboring tissues, adhesion to lymphatic and venous endothelia and immune escape. The existing functional and clinical background is now sufficiently solid to support interventive actions towards clinical applications.

The sweet side to cancer-associated glycans sour end resides on the fact that they may dramatically alter the protein landscape, originating unique cancer fingerprints at the cell-surface, as elegantly demonstrated by several studies 20, 24, 26, 172. In fact, STn and SLe^A^ are not tumor-specific antigens but exhibit a sufficiently restricted expression pattern in healthy tissues to support biomarker potential. Their reflection in the cancer proteome may, however, provide the necessary path towards cancer neoantigens. The challenge is set on the identification of cancer specific STn and SLe^A^ glycopeptide moieties, thus overcoming limitations associated with the presence of these glycans in low abundance in non-malignant conditions (**Figure 8**). Nevertheless, comprehensive targeted studies on the tumor STn and SLe^A-^glycoproteomes have not yet started and currently constitute an outstanding challenge to the field that must be addressed soon. Moreover, approaches to the serum glycoproteome have been of explorative nature and did not yet provided clinically translatable glycobiomarkers. Nevertheless, revisiting the circulating glycoproteins carrying these PTM in the context of health and disease will be crucial to better understand tumor development and decisive for improved serological tests. Moreover, the identification of functional glycoepitopes of clinical relevance will require the incorporation of glycoproteomics with proteogenomics perspectives, has elegantly highlighted for diffuse type GC (**Figure 8**). Other outstanding challenges facing precision medicine include the identification of glycosignatures to predict treatment outcome. In fact, a careful mapping of relevant cancer cell receptors such as ErbB2, PD-L1 or VEGFR2, which are glycoproteins targeted by therapeutic antibodies, may be critical to define resistance to therapeutics by impaired recognition. For instance, according to Duarte *et al.*, ErbB2, targeted by trastuzumab, expresses the SLe^A^ antigen in GC and cellular deglycosylation or antibody blocking of this glycan significantly altered ErbB2 expression and activation *in vitro*, suggesting that a better understanding of this receptor's glycosylation might be crucial for improving targeted intervention 20. Moreover, it has been demonstrated that altered PD-L1 glycosylation defines its recognition by PD-1 163 and this knowledge may be of key importance for selecting patient better served by this immunotherapy or improving current targeting. In a broader sense, alterations in protein glycocode are determinant for defining its immunogenicity and ultimately the cell metastatic potential by enabling immune escape. It is well established that some subsets of glycoproteins carrying STn and SLe^A^ may be recognized by siglecs and selectins on antigen presenting cells and lymphocytes 14, eliciting inhibitory signaling cascades. The identification of these glycoproteins may constitute an important milestone towards novel classes of immune check-point inhibitors. Some reports also supported that STn is expressed by chemoresistant cells presenting stem-cell phenotypes 204, and functional studies have directly implicated STn in this process 56. Deeper understanding of the chemoresistance- associated glycoproteome will be crucial for defining patients better served by alternative therapeutics and designing therapeutic alternatives.

In the context of therapeutics, important pre-clinical studies and clinical trials have explored glycans with exciting up-front results. The approaches are diverse, including glycovaccines, targeted nanovehicles for controlled drug delivery, bi-specific antibodies targeting specific glycodomains in relevant glycoproteins, and CAR-T cells directed against glycoepitopes, with emphasis on the STn antigen (**Figure 8**). Antagonists and inhibitors are also available to selectively inhibit glycan biosynthesis, and a wide array of strategies interfere with glycan-lectin interactions, which are central axes of many aspects of cancer progression, including cell proliferation, invasion, extravasation and immune evasion. However, the efficacy of targeted therapeutics focusing on cell-surface glycans remains challenged by the lack of target specificity, making true advances in this field dependent on the reinvestigation of the glycoproteome. More effective immunotherapies will also require a deeper understanding about the mechanisms by which glycans mediate immune tolerance, including the specific nature of the glycoproteins and the full repertoire of immune cells lectins involved in these processes.

In summary, classical glycobiomarkers have served us well in serological settings but still hold a tremendous unexplored potential to improve gastroesophageal and CRC patient's management, regarding that outstanding, yet achievable, milestones are accomplished. The current rationale supports the need to engage in glycan-targeted glycoproteomics screening of large and well characterized patient sets from both the molecular and clinicopathological standpoints envisaging cancer glyconeoantigens (**Figure 8**). The success of this approach is directly linked to a better understanding of the human STn and SLe^A^-glycoproteome in health and disease and its spatio-temporal evolution over the course of disease management and in response to microenvironmental cues. Reinvestigating the human glycoproteome is particularly critical since our current vision of glycosylation is, to some extent, blurred by the information retrieved from immunoassays based on a diversified array of antibodies with distinct affinities for glycan chains. The known implications of both STn and SLe^A^ in disease progression also vow for an emphasis on the molecular characterization of the metastasis from this angle. Nevertheless, it is critical to progress beyond proof-of-concept approaches regarding the inclusion of glycoproteomics into multi-omics biomarker research. According to recent findings, this step will be crucial for establishing more comprehensive patient stratification models, the identification of potentially targetable glycobiomarkers and the rationale design of targeted therapeutics.

## Figures and Tables

**Table 1:** Correlation between SLe^A^ and STn tissue expression and clinicopathologic variables of gastroesophageal and colorectal tumors.

Tumor Type	Antigen/ Antibody	N	Outcome	Ref
**Esophageal Cancer (SCC)**	SLe^A^ /NS19-9	54	-	111
	SLe^A^ /CA19.9	74	Tumor differentiation (P < 0.05) Poor prognosis (P < 0.05)	112
	SLe^A^ /Anti-SLe^A^	125	Hematogenous recurrence (p=0.026) Distant lymph node metastasis	113
	STn/ B72.3	84	-	94
	SLe^A^ /CA19.9	115	Stromal type of staining: Depth of invasion Tumor size	114
	SLe^A^ /CA19.9	52	Stromal type of staining: Peritoneal dissemination (P < 0.05) Lymphatic invasion (P < 0.05)	96
	STn/ B72.3 and TKH2	52	Stromal type of staining: Peritoneal dissemination in undifferentiated tumors (P < 0.05)	96
**Gastric cancer**	STn/ C 1282	242	Overall survival of stage I patients (P= 0.002) Prognosis (P=0.005)	97
	STn/ HB-STn1	211	Venous invasion (P < 0.05) Overall survival (P < 0.05) Prognosis (P=0.042)	98
	STn/ TKH2	60	Depth of invasion (P< 0.01) Tumor stage (P< 0.01) Lymph node metastasis (P< 0.005) Lymphatic invasion (P< 0.05) 5-year survival of advanced cases (P< 0.01)	58
	STn/ TKH2	54	-	99
	SLe^A^/CSLEA1	62	-	119
	SLe^A^ /NS19-9	309	Lymph node metastasis (P < 0.005) Recurrence (P < 0.005) Postoperative survival (P < 0.001	116
	SLe^A^ /CA19.9	52	-	117
	Unspecified	149	Histological type (P< 0.05) Depth of invasion (P< 0.05) Lymph node metastasis (P< 0.05) Venous invasion (P< 0.05) Astler-Coller stage (P< 0.05) TNM stage (P< 0.05) Recurrence (P< 0.01) 5-year survival (P< 0.01)	115
	SLe^A^ /NS19-9	52	5-year survival (P< 0.05)	110
**Colorectal cancer**	SLe^A^ /NS19-9	368	5-year disease-free survival (P=0.012) Recurrence	118
	STn/ TKH2	52	-	110
	STn/ TKH2	142	Depth of invasion	99
	STn/ HB-STn1	152	TNM classification (P=0.025) AJCC classification (P=0.049) in transitional tissue	103
	STn/ B72.3	234	Histological differentiation (P=0.006) Perineural invasion (P=0.041)	104
	STn/ C 1282	239	Age (P=0.024)	105
	STn/ TKH2	111	-	106

-No correlation to clinicopathological variables was found

## References

[1] Bray F, Ferlay J, Soerjomataram I, Siegel RL, Torre LA, Jemal A (2018). Global cancer statistics 2018: GLOBOCAN estimates of incidence and mortality worldwide for 36 cancers in 185 countries. CA Cancer J Clin.

[2] Flejou JF (2011). [WHO Classification of digestive tumors: the fourth edition]. Ann Pathol.

[3] Bijlsma MF, Sadanandam A, Tan P, Vermeulen L (2017). Molecular subtypes in cancers of the gastrointestinal tract. Nature Reviews Gastroenterology &Amp; Hepatology.

[4] Ross-Innes CS, Becq J, Warren A, Cheetham RK, Northen H, O'Donovan M (2015). Whole-genome sequencing provides new insights into the clonal architecture of Barrett's esophagus and esophageal adenocarcinoma. Nature Genetics.

[5] Molinari C, Marisi G, Passardi A, Matteucci L, De Maio G, Ulivi P (2018). Heterogeneity in Colorectal Cancer: A Challenge for Personalized Medicine?. Int J Mol Sci.

[6] Peinado H, Zhang H, Matei IR, Costa-Silva B, Hoshino A, Rodrigues G (2017). Pre-metastatic niches: organ-specific homes for metastases. Nat Rev Cancer.

[7] Allum WH, Blazeby JM, Griffin SM, Cunningham D, Jankowski JA, Wong R (2011). Guidelines for the management of esophageal and gastric cancer. Gut.

[8] Van der Jeught K, Xu HC, Li YJ, Lu XB, Ji G (2018). Drug resistance and new therapies in colorectal cancer. World J Gastroenterol.

[9] Kirstein MM, Lange A, Prenzler A, Manns MP, Kubicka S, Vogel A (2014). Targeted therapies in metastatic colorectal cancer: a systematic review and assessment of currently available data. Oncologist.

[10] Togasaki K, Sukawa Y, Kanai T, Takaishi H (2018). Clinical efficacy of immune checkpoint inhibitors in the treatment of unresectable advanced or recurrent gastric cancer: an evidence-based review of therapies. Onco Targets Ther.

[11] Visnovska T, Biggs PJ, Schmeier S, Frizelle FA, Purcell RV (2019). Metagenomics and transcriptomics data from human colorectal cancer. Scientific Data.

[12] Ge S, Xia X, Ding C, Zhen B, Zhou Q, Feng J (2018). A proteomic landscape of diffuse-type gastric cancer. Nature Communications.

[13] Vasaikar S, Huang C, Wang X, Petyuk VA, Savage SR, Wen B (2019). Proteogenomic Analysis of Human Colon Cancer Reveals New Therapeutic Opportunities. Cell.

[14] Peixoto A, Relvas-Santos M, Azevedo R, Santos LL, Ferreira JA (2019). Protein Glycosylation and Tumor Microenvironment Alterations Driving Cancer Hallmarks. Front Oncol.

[15] Azevedo R, Peixoto A, Gaiteiro C, Fernandes E, Neves M, Lima L (2017). Over forty years of bladder cancer glycobiology: Where do glycans stand facing precision oncology?. Oncotarget.

[16] Marcos NT, Bennett EP, Gomes J, Magalhaes A, Gomes C, David L (2011). ST6GalNAc-I controls expression of sialyl-Tn antigen in gastrointestinal tissues. Front Biosci (Elite Ed).

[17] Reis CA, Osorio H, Silva L, Gomes C, David L (2010). Alterations in glycosylation as biomarkers for cancer detection. J Clin Pathol.

[18] Ferreira JA, Magalhaes A, Gomes J, Peixoto A, Gaiteiro C, Fernandes E (2017). Protein glycosylation in gastric and colorectal cancers: Toward cancer detection and targeted therapeutics. Cancer Lett.

[19] Pinho S, Marcos NT, Ferreira B, Carvalho AS, Oliveira MJ, Santos-Silva F (2007). Biological significance of cancer-associated sialyl-Tn antigen: modulation of malignant phenotype in gastric carcinoma cells. Cancer Lett.

[20] Duarte HO, Balmana M, Mereiter S, Osorio H, Gomes J, Reis CA (2017). Gastric Cancer Cell Glycosylation as a Modulator of the ErbB2 Oncogenic Receptor. Int J Mol Sci.

[21] Pinho SS, Reis CA (2015). Glycosylation in cancer: mechanisms and clinical implications. Nat Rev Cancer.

[22] Ferreira IG, Pucci M, Venturi G, Malagolini N, Chiricolo M, Dall'Olio F (2018). Glycosylation as a Main Regulator of Growth and Death Factor Receptors Signaling. Int J Mol Sci.

[23] Pinho SS, Carvalho S, Marcos-Pinto R, Magalhaes A, Oliveira C, Gu J (2013). Gastric cancer: adding glycosylation to the equation. Trends Mol Med.

[24] Mereiter S, Martins AM, Gomes C, Balmana M, Macedo JA, Polom K (2019). O-glycan truncation enhances cancer-related functions of CD44 in gastric cancer. FEBS Lett.

[25] RodrÍguez E, Schetters STT, van Kooyk Y (2018). The tumor glyco-code as a novel immune checkpoint for immunotherapy. Nature Reviews Immunology.

[26] Peixoto A, Fernandes E, Gaiteiro C, Lima L, Azevedo R, Soares J (2016). Hypoxia enhances the malignant nature of bladder cancer cells and concomitantly antagonizes protein O-glycosylation extension. Oncotarget.

[27] Gill DJ, Clausen H, Bard F (2011). Location, location, location: new insights into O-GalNAc protein glycosylation. Trends Cell Biol.

[28] Brockhausen I, Stanley P (2015). O-GalNAc Glycans. In: rd, Varki A, Cummings RD, Esko JD, Stanley P, Hart GW, et al. Essentials of Glycobiology. Cold Spring Harbor (NY).

[29] Duarte HO, Freitas D, Gomes C, Gomes J, Magalhaes A, Reis CA (2016). Mucin-Type O-Glycosylation in Gastric Carcinogenesis. Biomolecules.

[30] Shiozaki K, Yamaguchi K, Takahashi K, Moriya S, Miyagi T (2011). Regulation of sialyl Lewis antigen expression in colon cancer cells by sialidase NEU4. J Biol Chem.

[31] Julien S, Videira PA, Delannoy P (2012). Sialyl-tn in cancer: (how) did we miss the target?. Biomolecules.

[32] Marcos NT, Pinho S, Grandela C, Cruz A, Samyn-Petit B, Harduin-Lepers A (2004). Role of the human ST6GalNAc-I and ST6GalNAc-II in the synthesis of the cancer-associated sialyl-Tn antigen. Cancer Res.

[33] Ju T, Lanneau GS, Gautam T, Wang Y, Xia B, Stowell SR (2008). Human tumor antigens Tn and sialyl Tn arise from mutations in Cosmc. Cancer Res.

[34] Radhakrishnan P, Dabelsteen S, Madsen FB, Francavilla C, Kopp KL, Steentoft C (2014). Immature truncated O-glycophenotype of cancer directly induces oncogenic features. Proc Natl Acad Sci U S A.

[35] Schultz MJ, Swindall AF, Bellis SL (2012). Regulation of the metastatic cell phenotype by sialylated glycans. Cancer Metastasis Rev.

[36] Gill DJ, Tham KM, Chia J, Wang SC, Steentoft C, Clausen H (2013). Initiation of GalNAc-type O-glycosylation in the endoplasmic reticulum promotes cancer cell invasiveness. Proc Natl Acad Sci U S A.

[37] Kudelka MR, Ju T, Heimburg-Molinaro J, Cummings RD (2015). Simple sugars to complex disease-mucin-type O-glycans in cancer. Adv Cancer Res.

[38] Steentoft C, Vakhrushev SY, Joshi HJ, Kong Y, Vester-Christensen MB, Schjoldager KT (2013). Precision mapping of the human O-GalNAc glycoproteome through SimpleCell technology. EMBO J.

[39] Loureiro LR, Feldmann A, Bergmann R, Koristka S, Berndt N, Arndt C (2018). Development of a novel target module redirecting UniCAR T cells to Sialyl Tn-expressing tumor cells. Blood Cancer J.

[40] Dall'Olio F, Malagolini N, Trinchera M, Chiricolo M (2012). Mechanisms of cancer-associated glycosylation changes. Front Biosci (Landmark Ed).

[41] Gomes C, Almeida A, Barreira A, Calheiros J, Pinto F, Abrantes R (2019). Carcinoembryonic antigen carrying SLe(X) as a new biomarker of more aggressive gastric carcinomas. Theranostics.

[42] Shimodaira K, Nakayama J, Nakamura N, Hasebe O, Katsuyama T, Fukuda M (1997). Carcinoma-associated expression of core 2 beta-1,6-N-acetylglucosaminyltransferase gene in human colorectal cancer: role of O-glycans in tumor progression. Cancer Res.

[43] Holmes EH, Hakomori S, Ostrander GK (1987). Synthesis of type 1 and 2 lacto series glycolipid antigens in human colonic adenocarcinoma and derived cell lines is due to activation of a normally unexpressed beta 1--3N-acetylglucosaminyltransferase. J Biol Chem.

[44] Trinchera M, Aronica A, Dall'Olio F (2017). Selectin Ligands Sialyl-Lewis a and Sialyl-Lewis x in Gastrointestinal Cancers. Biology (Basel).

[45] Itai S, Nishikata J, Yoneda T, Ohmori K, Yamabe H, Arii S (1991). Tissue distribution of 2-3 and 2-6 sialyl Lewis A antigens and significance of the ratio of two antigens for the differential diagnosis of malignant and benign disorders of the digestive tract. Cancer.

[46] Miyazaki K, Sakuma K, Kawamura YI, Izawa M, Ohmori K, Mitsuki M (2012). Colonic epithelial cells express specific ligands for mucosal macrophage immunosuppressive receptors siglec-7 and -9. J Immunol.

[47] Miyazaki K, Ohmori K, Izawa M, Koike T, Kumamoto K, Furukawa K (2004). Loss of disialyl Lewis(a), the ligand for lymphocyte inhibitory receptor sialic acid-binding immunoglobulin-like lectin-7 (Siglec-7) associated with increased sialyl Lewis(a) expression on human colon cancers. Cancer Res.

[48] Izawa M, Kumamoto K, Mitsuoka C, Kanamori C, Kanamori A, Ohmori K (2000). Expression of sialyl 6-sulfo Lewis X is inversely correlated with conventional sialyl Lewis X expression in human colorectal cancer. Cancer Res.

[49] Yusa A, Miyazaki K, Kimura N, Izawa M, Kannagi R (2010). Epigenetic silencing of the sulfate transporter gene DTDST induces sialyl Lewisx expression and accelerates proliferation of colon cancer cells. Cancer Res.

[50] Kannagi R, Sakuma K, Miyazaki K, Lim KT, Yusa A, Yin J (2010). Altered expression of glycan genes in cancers induced by epigenetic silencing and tumor hypoxia: clues in the ongoing search for new tumor markers. Cancer Sci.

[51] Ikeda Y, Kuwano H, Baba K, Ikebe M, Matushima T, Adachi Y (1993). Expression of Sialyl-Tn antigens in normal squamous epithelium, dysplasia, and squamous cell carcinoma in the esophagus. Cancer Res.

[52] Ohuchi N, Thor A, Nose M, Fujita J, Kyogoku M, Schlom J (1986). Tumor-associated glycoprotein (TAG-72) detected in adenocarcinomas and benign lesions of the stomach. Int J Cancer.

[53] Itzkowitz SH, Yuan M, Montgomery CK, Kjeldsen T, Takahashi HK, Bigbee WL (1989). Expression of Tn, sialosyl-Tn, and T antigens in human colon cancer. Cancer Res.

[54] Freitas D, Campos D, Gomes J, Pinto F, Macedo JA, Matos R (2019). O-glycans truncation modulates gastric cancer cell signaling and transcription leading to a more aggressive phenotype. EBioMedicine.

[55] Ozaki H, Matsuzaki H, Ando H, Kaji H, Nakanishi H, Ikehara Y (2012). Enhancement of metastatic ability by ectopic expression of ST6GalNAcI on a gastric cancer cell line in a mouse model. Clin Exp Metastasis.

[56] Santos SN, Junqueira MS, Francisco G, Vilanova M, Magalhaes A, Dias Baruffi M (2016). O-glycan sialylation alters galectin-3 subcellular localization and decreases chemotherapy sensitivity in gastric cancer. Oncotarget.

[57] Neves M, Azevedo R, Lima L, Oliveira MI, Peixoto A, Ferreira D (2019). Exploring sialyl-Tn expression in microfluidic-isolated circulating tumor cells: A novel biomarker and an analytical tool for precision oncology applications. N Biotechnol.

[58] Yamada T, Watanabe A, Yamada Y, Shino Y, Tanase M, Yamashita J (1995). Sialosyl Tn antigen expression is associated with the prognosis of patients with advanced gastric cancer. Cancer.

[59] Lima L, Neves M, Oliveira MI, Dieguez L, Freitas R, Azevedo R (2017). Sialyl-Tn identifies muscle-invasive bladder cancer basal and luminal subtypes facing decreased survival, being expressed by circulating tumor cells and metastases. Urol Oncol.

[60] Ogawa T, Hirohashi Y, Murai A, Nishidate T, Okita K, Wang L (2017). ST6GALNAC1 plays important roles in enhancing cancer stem phenotypes of colorectal cancer via the Akt pathway. Oncotarget.

[61] Singh R, Campbell BJ, Yu LG, Fernig DG, Milton JD, Goodlad RA (2001). Cell surface-expressed Thomsen-Friedenreich antigen in colon cancer is predominantly carried on high molecular weight splice variants of CD44. Glycobiology.

[62] Munkley J (2016). The Role of Sialyl-Tn in Cancer. Int J Mol Sci.

[63] Zhang H, Li H, Ma Q, Yang FY, Diao TY (2016). Predicting malignant transformation of esophageal squamous cell lesions by combined biomarkers in an endoscopic screening program. World J Gastroenterol.

[64] Futamura N, Nakamura S, Tatematsu M, Yamamura Y, Kannagi R, Hirose H (2000). Clinicopathologic significance of sialyl Le(x) expression in advanced gastric carcinoma. Br J Cancer.

[65] Takada A, Ohmori K, Yoneda T, Tsuyuoka K, Hasegawa A, Kiso M (1993). Contribution of carbohydrate antigens sialyl Lewis A and sialyl Lewis X to adhesion of human cancer cells to vascular endothelium. Cancer Res.

[66] Ito K, Ye CL, Hibi K, Mitsuoka C, Kannagi R, Hidemura K (2001). Paired tumor marker of soluble E-selectin and its ligand sialyl Lewis A in colorectal cancer. Journal of gastroenterology.

[67] Shimada Y, Maeda M, Watanabe G, Imamura M (2003). High serum soluble E-selectin levels are associated with postoperative haematogenic recurrence in esophageal squamous cell carcinoma patients. Oncol Rep.

[68] Kannagi R, Izawa M, Koike T, Miyazaki K, Kimura N (2004). Carbohydrate-mediated cell adhesion in cancer metastasis and angiogenesis. Cancer Sci.

[69] McCarty OJ, Mousa SA, Bray PF, Konstantopoulos K (2000). Immobilized platelets support human colon carcinoma cell tethering, rolling, and firm adhesion under dynamic flow conditions. Blood.

[70] Gay LJ, Felding-Habermann B (2011). Contribution of platelets to tumor metastasis. Nature Reviews Cancer.

[71] Honn KV, Tang DG, Crissman JD (1992). Platelets and cancer metastasis: a causal relationship?. Cancer Metastasis Rev.

[72] Sakuma K, Aoki M, Kannagi R (2012). Transcription factors c-Myc and CDX2 mediate E-selectin ligand expression in colon cancer cells undergoing EGF/bFGF-induced epithelial-mesenchymal transition. Proc Natl Acad Sci U S A.

[73] Takahashi M, Yokoe S, Asahi M, Lee SH, Li W, Osumi D (2008). N-glycan of ErbB family plays a crucial role in dimer formation and tumor promotion. Biochim Biophys Acta.

[74] Marcos NT, Magalhaes A, Ferreira B, Oliveira MJ, Carvalho AS, Mendes N (2008). Helicobacter pylori induces beta3GnT5 in human gastric cell lines, modulating expression of the SabA ligand sialyl-Lewis x. J Clin Invest.

[75] Guadagni F, Roselli M, Amato T, Cosimelli M, Perri P, Casale V (1992). CA 72-4 measurement of tumor-associated glycoprotein 72 (TAG-72) as a serum marker in the management of gastric carcinoma. Cancer Res.

[76] Magnani JL, Steplewski Z, Koprowski H, Ginsburg V (1983). Identification of the gastrointestinal and pancreatic cancer-associated antigen detected by monoclonal antibody 19-9 in the sera of patients as a mucin. Cancer Res.

[77] Song YX, Huang XZ, Gao P, Sun JX, Chen XW, Yang YC (2015). Clinicopathologic and Prognostic Value of Serum Carbohydrate Antigen 19-9 in Gastric Cancer: A Meta-Analysis. Dis Markers.

[78] Shimada H, Noie T, Ohashi M, Oba K, Takahashi Y (2014). Clinical significance of serum tumor markers for gastric cancer: a systematic review of literature by the Task Force of the Japanese Gastric Cancer Association. Gastric Cancer.

[79] Hu PJ, Chen MY, Wu MS, Lin YC, Shih PH, Lai CH (2019). Clinical Evaluation of CA72-4 for Screening Gastric Cancer in A Healthy Population: A Multicenter Retrospective Study. Cancers (Basel).

[80] Xu Y, Chen Y, Wei L, Lai S, Zheng W, Wu F (2019). Serum tumor-associated glycoprotein 72, a helpful predictor of lymph nodes invasion in esophagogastric junction adenocarcinoma. Biochem Biophys Res Commun.

[81] Kannagi R (2007). Carbohydrate antigen sialyl Lewis a-its pathophysiological significance and induction mechanism in cancer progression. Chang Gung Med J.

[82] Yu Z, Chen Z, Wu J, Li Z, Wu Y (2017). Prognostic value of pretreatment serum carbohydrate antigen 19-9 level in patients with colorectal cancer: A meta-analysis. PLoS One.

[83] Gao Y, Wang J, Zhou Y, Sheng S, Qian SY, Huo X (2018). Evaluation of Serum CEA, CA19-9, CA72-4, CA125 and Ferritin as Diagnostic Markers and Factors of Clinical Parameters for Colorectal Cancer. Sci Rep.

[84] Yanqing H, Cheng D, Ling X (2018). Serum CA72-4 as a Biomarker in the Diagnosis of Colorectal Cancer: A Meta-analysis. Open Med (Wars).

[85] Zhao H, Chen W, Wu J, Wang L, Mao W (2014). Clinical significance of preoperative serum tumor markers in esophageal squamous cell carcinoma. Journal of Cancer Research and Therapeutics.

[86] Bagaria B, Bagaria A, Singh M, Sharma R (2015). Diagnostic sensitivity of serum carcinoembryonic antigen, carbohydrate antigen 19-9, alpha-fetoprotein, and beta-human chorionic gonadotropin in esophageal carcinoma (receiver operating characteristic curve analysis). Clinical Cancer Investigation Journal.

[87] Azevedo R, Soares J, Peixoto A, Cotton S, Lima L, Santos LL (2018). Circulating tumor cells in bladder cancer: Emerging technologies and clinical implications foreseeing precision oncology. Urol Oncol.

[88] Alunni-Fabbroni M, Muller V, Fehm T, Janni W, Rack B (2014). Monitoring in metastatic breast cancer: is imaging outdated in the era of circulating tumor cells?. Breast Care (Basel).

[89] Grolz D, Hauch S, Schlumpberger M, Guenther K, Voss T, Sprenger-Haussels M (2018). Liquid Biopsy Preservation Solutions for Standardized Pre-Analytical Workflows-Venous Whole Blood and Plasma. Curr Pathobiol Rep.

[90] Hofman V, Heeke S, Marquette CH, Ilie M, Hofman P (2019). Circulating Tumor Cell Detection in Lung Cancer: But to What End?. Cancers (Basel).

[91] Lopresti A, Malergue F, Bertucci F, Liberatoscioli ML, Garnier S, DaCosta Q (2019). Sensitive and easy screening for circulating tumor cells by flow cytometry. JCI Insight.

[92] Nuti M, Teramoto YA, Mariani-Costantini R, Hand PH, Colcher D, Schlom J (1982). A monoclonal antibody (B72.3) defines patterns of distribution of a novel tumor-associated antigen in human mammary carcinoma cell populations. Int J Cancer.

[93] Reddish MA, Jackson L, Koganty RR, Qiu D, Hong W, Longenecker BM (1997). Specificities of anti-sialyl-Tn and anti-Tn monoclonal antibodies generated using novel clustered synthetic glycopeptide epitopes. Glycoconj J.

[94] Flucke U, Zirbes TK, Schroder W, Monig SP, Koch V, Schmitz K (2001). Expression of mucin-associated carbohydrate core antigens in esophageal squamous cell carcinomas. Anticancer Res.

[95] Thor A, Ohuchi N, Szpak CA, Johnston WW, Schlom J (1986). Distribution of oncofetal antigen tumor-associated glycoprotein-72 defined by monoclonal antibody B72.3. Cancer Res.

[96] Ikeda Y, Mori M, Kamakura T, Saku M, Sugimachi K (1995). Immunohistochemical expression of sialyl Tn and sialyl Lewis(a) antigens in stromal tissue correlates with peritoneal dissemination in stage IV human gastric cancer. Eur J Surg Oncol.

[97] Victorzon M, Nordling S, Nilsson O, Roberts PJ, Haglund C (1996). Sialyl Tn antigen is an independent predictor of outcome in patients with gastric cancer. Int J Cancer.

[98] Terashima S, Takano Y, Ohori T, Kanno T, Kimura T, Motoki R (1998). Sialyl-Tn antigen as a useful predictor of poor prognosis in patients with advanced stomach cancer. Surg Today.

[99] Yamashita Y, Chung YS, Sawada T, Horie R, Saito T, Murayama K (1998). F1 alpha: a novel mucin antigen associated with gastric carcinogenesis. Oncology.

[100] Gomes C, Almeida A, Ferreira JA, Silva L, Santos-Sousa H, Pinto-de-Sousa J (2013). Glycoproteomic analysis of serum from patients with gastric precancerous lesions. J Proteome Res.

[101] Kakeji Y, Maehara Y, Morita M, Matsukuma A, Furusawa M, Takahashi I (1995). Correlation between sialyl Tn antigen and lymphatic metastasis in patients with Borrmann type IV gastric carcinoma. Br J Cancer.

[102] Yamachika T, Nakanishi H, Inada K, Kitoh K, Kato T, Irimura T (1997). Reciprocal control of colon-specific sulfomucin and sialosyl-Tn antigen expression in human colorectal neoplasia. Virchows Arch.

[103] Vazquez-Martin C, Cuevas E, Gil-Martin E, Fernandez-Briera A (2004). Correlation analysis between tumor-associated antigen sialyl-Tn expression and ST6GalNAc I activity in human colon adenocarcinoma. Oncology.

[104] Xu F, Fan C, Fan S, Liu F, Wen T, An G (2015). Expression profile of mucin-associated sialyl-Tn antigen in Chinese patients with different colorectal lesions (adenomas, carcinomas). Int J Clin Exp Pathol.

[105] Lundin M, Nordling S, Roberts PJ, Lundin J, Carpelan-Holmstrom M, von Boguslawsky K (1999). Sialyl Tn is a frequently expressed antigen in colorectal cancer: No correlation with patient prognosis. Oncology.

[106] Nanashima A, Nakagoe T, Sawai T, Nakamura S, Yamaguchi H, Yasutake T (1997). Different expressions of sialyl Tn antigen between polypoid and flat-type early colorectal cancers. Dis Colon Rectum.

[107] Kjeldsen T, Clausen H, Hirohashi S, Ogawa T, Iijima H, Hakomori S (1988). Preparation and characterization of monoclonal antibodies directed to the tumor-associated O-linked sialosyl-2--6 alpha-N-acetylgalactosaminyl (sialosyl-Tn) epitope. Cancer Res.

[108] Ogata S, Koganty R, Reddish M, Longenecker BM, Chen A, Perez C (1998). Different modes of sialyl-Tn expression during malignant transformation of human colonic mucosa. Glycoconj J.

[109] Shen Y, Tiralongo J, Kohla G, Schauer R (2004). Regulation of sialic acid O-acetylation in human colon mucosa. Biol Chem.

[110] Akamine S, Nakagoe T, Sawai T, Tsuji T, Tanaka K, Hidaka S (2004). Differences in prognosis of colorectal cancer patients based on the expression of sialyl Lewisa, sialyl Lewisx and sialyl Tn antigens in serum and tumor tissue. Anticancer Res.

[111] Oshiba G, Kijima H, Tanaka H, Kenmochi T, Himeno S, Kise Y (2000). Frequent expression of sialyl Le(a) in human esophageal squamous cell carcinoma. Int J Oncol.

[112] Ikeda Y, Kuwano H, Ikebe M, Baba K, Toh Y, Adachi Y (1994). Immunohistochemical detection of CEA, CA19-9, and DF3 in esophageal carcinoma limited to the submucosal layer. J Surg Oncol.

[113] Makino T, Shimada Y, Maeda M, Komoto I, Imamura M (2001). Carbohydrate antigens as a risk factor for hematogenous recurrence of esophageal squamous cell carcinoma patients. Oncol Rep.

[114] Ikeda Y, Mori M, Kido A, Shimono R, Matsushima T, Sugimachi K (1991). Immunohistochemical expression of carbohydrate antigen 19-9 in gastric carcinoma. Am J Gastroenterol.

[115] Shimono R, Mori M, Akazawa K, Adachi Y, Sgimachi K (1994). Immunohistochemical expression of carbohydrate antigen 19-9 in colorectal carcinoma. Am J Gastroenterol.

[116] Nakayama T, Watanabe M, Katsumata T, Teramoto T, Kitajima M (1995). Expression of sialyl Lewis(a) as a new prognostic factor for patients with advanced colorectal carcinoma. Cancer.

[117] Lorenzi M, Vindigni C, Minacci C, Tripodi SA, Iroatulam A, Petrioli R (1997). Histopathological and prognostic evaluation of immunohistochemical findings in colorectal cancer. Int J Biol Markers.

[118] Portela SV, Martin CV, Romay LM, Cuevas E, Martin EG, Briera AF (2011). sLea and sLex expression in colorectal cancer: implications for tumorigenesis and disease prognosis. Histol Histopathol.

[119] Nakagoe T, Kusano H, Hirota M, Fukushima K, Hiratani K, Hara K (1991). Serological and immunohistochemical studies on sialylated carbohydrate antigens in colorectal carcinoma. Gastroenterol Jpn.

[120] Nakayama T, Watanabe M, Teramoto T, Kitajima M (1997). CA19-9 as a predictor of recurrence in patients with colorectal cancer. J Surg Oncol.

[121] Magnani JL, Nilsson B, Brockhaus M, Zopf D, Steplewski Z, Koprowski H (1982). A monoclonal antibody-defined antigen associated with gastrointestinal cancer is a ganglioside containing sialylated lacto-N-fucopentaose II. J Biol Chem.

[122] Manne U, Weiss HL, Myers RB, Danner OK, Moron C, Srivastava S (1998). Nuclear accumulation of p53 in colorectal adenocarcinoma: prognostic importance differs with race and location of the tumor. Cancer.

[123] Manne U, Weiss HL, Grizzle WE (2000). Racial differences in the prognostic usefulness of MUC1 and MUC2 in colorectal adenocarcinomas. Clin Cancer Res.

[124] Mereiter S, Balmana M, Campos D, Gomes J, Reis CA (2019). Glycosylation in the Era of Cancer-Targeted Therapy: Where Are We Heading?. Cancer Cell.

[125] O'Cearbhaill RE, Ragupathi G, Zhu J, Wan Q, Mironov S, Yang G (2016). A Phase I Study of Unimolecular Pentavalent (Globo-H-GM2-sTn-TF-Tn) Immunization of Patients with Epithelial Ovarian, Fallopian Tube, or Peritoneal Cancer in First Remission. Cancers (Basel).

[126] Miles D, Roche H, Martin M, Perren TJ, Cameron DA, Glaspy J (2011). Phase III multicenter clinical trial of the sialyl-TN (STn)-keyhole limpet hemocyanin (KLH) vaccine for metastatic breast cancer. Oncologist.

[127] Ibrahim NK, Murray JL, Zhou D, Mittendorf EA, Sample D, Tautchin M (2013). Survival Advantage in Patients with Metastatic Breast Cancer Receiving Endocrine Therapy plus Sialyl Tn-KLH Vaccine: Post Hoc Analysis of a Large Randomized Trial. J Cancer.

[128] Zeichner SB (2012). The Failed Theratope Vaccine: 10 Years Later. The Journal of the American Osteopathic Association.

[129] Pedersen JW, Blixt O, Bennett EP, Tarp MA, Dar I, Mandel U (2011). Seromic profiling of colorectal cancer patients with novel glycopeptide microarray. Int J Cancer.

[130] Carrascal MA, Severino PF, Guadalupe Cabral M, Silva M, Ferreira JA, Calais F (2014). Sialyl Tn-expressing bladder cancer cells induce a tolerogenic phenotype in innate and adaptive immune cells. Mol Oncol.

[131] Rughetti A, Pellicciotta I, Biffoni M, Backstrom M, Link T, Bennet EP (2005). Recombinant tumor-associated MUC1 glycoprotein impairs the differentiation and function of dendritic cells. J Immunol.

[132] Ninkovic T, Hanisch FG (2007). O-glycosylated human MUC1 repeats are processed in vitro by immunoproteasomes. J Immunol.

[133] Gaidzik N, Westerlind U, Kunz H (2013). The development of synthetic antitumor vaccines from mucin glycopeptide antigens. Chem Soc Rev.

[134] Abdel-Aal AB, Lakshminarayanan V, Thompson P, Supekar N, Bradley JM, Wolfert MA (2014). Immune and anticancer responses elicited by fully synthetic aberrantly glycosylated MUC1 tripartite vaccines modified by a TLR2 or TLR9 agonist. Chembiochem.

[135] Song C, Zheng XJ, Liu CC, Zhou Y, Ye XS (2017). A cancer vaccine based on fluorine-modified sialyl-Tn induces robust immune responses in a murine model. Oncotarget.

[136] Ragupathi G, Damani P, Srivastava G, Srivastava O, Sucheck SJ, Ichikawa Y (2009). Synthesis of sialyl Lewis(a) (sLe (a), CA19-9) and construction of an immunogenic sLe(a) vaccine. Cancer Immunol Immunother.

[137] Taylor-Papadimitriou J, Burchell JM, Graham R, Beatson R (2018). Latest developments in MUC1 immunotherapy. Biochem Soc Trans.

[138] Lakshminarayanan V, Supekar NT, Wei J, McCurry DB, Dueck AC, Kosiorek HE (2016). MUC1 Vaccines, Comprised of Glycosylated or Non-Glycosylated Peptides or Tumor-Derived MUC1, Can Circumvent Immunoediting to Control Tumor Growth in MUC1 Transgenic Mice. PLoS One.

[139] Reis CA, David L, Seixas M, Burchell J, Sobrinho-Simoes M (1998). Expression of fully and under-glycosylated forms of MUC1 mucin in gastric carcinoma. Int J Cancer.

[140] Wang Y, Liao X, Ye Q, Huang L (2018). Clinic implication of MUC1 O-glycosylation and C1GALT1 in esophagus squamous cell carcinoma. Sci China Life Sci.

[141] Stergiou N, Gaidzik N, Heimes AS, Dietzen S, Besenius P, Jakel J (2019). Reduced Breast Tumor Growth after Immunization with a Tumor-Restricted MUC1 Glycopeptide Conjugated to Tetanus Toxoid. Cancer Immunol Res.

[142] Hossain MK, Wall KA (2016). Immunological Evaluation of Recent MUC1 Glycopeptide Cancer Vaccines. Vaccines (Basel).

[143] Benmebarek MR, Karches CH, Cadilha BL, Lesch S, Endres S, Kobold S (2019). Killing Mechanisms of Chimeric Antigen Receptor (CAR) T Cells. Int J Mol Sci.

[144] Hombach A, Heuser C, Sircar R, Tillmann T, Diehl V, Kruis W (1997). T cell targeting of TAG72+ tumor cells by a chimeric receptor with antibody-like specificity for a carbohydrate epitope. Gastroenterology.

[145] Hege KM, Bergsland EK, Fisher GA, Nemunaitis JJ, Warren RS, McArthur JG (2017). Safety, tumor trafficking and immunogenicity of chimeric antigen receptor (CAR)-T cells specific for TAG-72 in colorectal cancer. J Immunother Cancer.

[146] Kim SJ, Hong HJ (2007). Guided selection of human antibody light chains against TAG-72 using a phage display chain shuffling approach. J Microbiol.

[147] Muraro R, Kuroki M, Wunderlich D, Poole DJ, Colcher D, Thor A (1988). Generation and characterization of B72.3 second generation monoclonal antibodies reactive with the tumor-associated glycoprotein 72 antigen. Cancer Res.

[148] Loureiro LR, Sousa DP, Ferreira D, Chai W, Lima L, Pereira C (2018). Novel monoclonal antibody L2A5 specifically targeting sialyl-Tn and short glycans terminated by alpha-2-6 sialic acids. Sci Rep.

[149] Itzkowitz SH, Young E, Dubois D, Harpaz N, Bodian C, Chen A (1996). Sialosyl-Tn antigen is prevalent and precedes dysplasia in ulcerative colitis: a retrospective case-control study. Gastroenterology.

[150] O'Boyle KP, Zamore R, Adluri S, Cohen A, Kemeny N, Welt S (1992). Immunization of colorectal cancer patients with modified ovine submaxillary gland mucin and adjuvants induces IgM and IgG antibodies to sialylated Tn. Cancer Res.

[151] Prendergast JM, Galvao da Silva AP, Eavarone DA, Ghaderi D, Zhang M, Brady D (2017). Novel anti-Sialyl-Tn monoclonal antibodies and antibody-drug conjugates demonstrate tumor specificity and anti-tumor activity. MAbs.

[152] Sawada R, Sun SM, Wu X, Hong F, Ragupathi G, Livingston PO (2011). Human monoclonal antibodies to sialyl-Lewis (CA19.9) with potent CDC, ADCC, and antitumor activity. Clin Cancer Res.

[153] Liu SD, Chalouni C, Young JC, Junttila TT, Sliwkowski MX, Lowe JB (2015). Afucosylated antibodies increase activation of FcgammaRIIIa-dependent signaling components to intensify processes promoting ADCC. Cancer Immunol Res.

[154] Loureiro LR, Carrascal MA, Barbas A, Ramalho JS, Novo C, Delannoy P (2015). Challenges in Antibody Development against Tn and Sialyl-Tn Antigens. Biomolecules.

[155] Seo Y, Ishii Y, Ochiai H, Fukuda K, Akimoto S, Hayashida T (2014). Cetuximab-mediated ADCC activity is correlated with the cell surface expression level of EGFR but not with the KRAS/BRAF mutational status in colorectal cancer. Oncol Rep.

[156] DeAngelo DJ, Jonas BA, Liesveld JL, Bixby DL, Advani AS, Marlton P (2017). GMI-1271 Improves Efficacy and Safety of Chemotherapy in R/R and Newly Diagnosed Older Patients with AML: Results of a Phase 1/2 Study. Blood.

[157] DeAngelo DJ, Erba HP, Jonas BA, O'Dwyer M, Marlton P, Huls GA (2019). A phase III trial to evaluate the efficacy of uproleselan (GMI-1271) with chemotherapy in patients with relapsed/refractory acute myeloid leukemia. Journal of Clinical Oncology.

[158] Alley SC, Meara M, Gardai SJ, Okeley NM (2017). Abstract DDT02-02: SGN-2FF: A novel small molecule inhibitor of fucosylation with preclinical antitumor activity through multiple immune mechanisms. Cancer Research.

[159] Zhou Y, Fukuda T, Hang Q, Hou S, Isaji T, Kameyama A (2017). Inhibition of fucosylation by 2-fluorofucose suppresses human liver cancer HepG2 cell proliferation and migration as well as tumor formation. Scientific Reports.

[160] Bull C, Boltje TJ, Balneger N, Weischer SM, Wassink M, van Gemst JJ (2018). Sialic Acid Blockade Suppresses Tumor Growth by Enhancing T-cell-Mediated Tumor Immunity. Cancer Res.

[161] Fernandes E, Ferreira JA, Andreia P, Luis L, Barroso S, Sarmento B (2015). New trends in guided nanotherapies for digestive cancers: A systematic review. J Control Release.

[162] Li CW, Lim SO, Chung EM, Kim YS, Park AH, Yao J (2018). Eradication of Triple-Negative Breast Cancer Cells by Targeting Glycosylated PD-L1. Cancer Cell.

[163] Hsu J-M, Li C-W, Lai Y-J, Hung M-C (2018). Posttranslational Modifications of PD-L1 and Their Applications in Cancer Therapy. Cancer Research.

[164] Hsu JM, Li CW, Lai YJ, Hung MC (2018). Posttranslational Modifications of PD-L1 and Their Applications in Cancer Therapy. Cancer Res.

[165] Tkac J, Gajdosova V, Hroncekova S, Bertok T, Hires M, Jane E (2019). Prostate-specific antigen glycoprofiling as diagnostic and prognostic biomarker of prostate cancer. Interface Focus.

[166] Llop E, Ferrer-Batalle M, Barrabes S, Guerrero PE, Ramirez M, Saldova R (2016). Improvement of Prostate Cancer Diagnosis by Detecting PSA Glycosylation-Specific Changes. Theranostics.

[167] Li QK, Chen L, Ao MH, Chiu JH, Zhang Z, Zhang H (2015). Serum fucosylated prostate-specific antigen (PSA) improves the differentiation of aggressive from non-aggressive prostate cancers. Theranostics.

[168] Saldova R, Struwe WB, Wynne K, Elia G, Duffy MJ, Rudd PM (2013). Exploring the glycosylation of serum CA125. Int J Mol Sci.

[169] Sola RJ, Griebenow K (2009). Effects of glycosylation on the stability of protein pharmaceuticals. J Pharm Sci.

[170] Nishikaze T (2017). Sensitive and Structure-Informative N-Glycosylation Analysis by MALDI-MS; Ionization, Fragmentation, and Derivatization. Mass Spectrom (Tokyo).

[171] Yang L, Sun Z, Zhang L, Cai Y, Peng Y, Cao T (2019). Chemical labeling for fine mapping of IgG N-glycosylation by ETD-MS. Chemical Science.

[172] Cotton S, Azevedo R, Gaiteiro C, Ferreira D, Lima L, Peixoto A (2017). Targeted O-glycoproteomics explored increased sialylation and identified MUC16 as a poor prognosis biomarker in advanced-stage bladder tumors. Mol Oncol.

[173] Mechref Y, Muddiman DC (2017). Recent advances in glycomics, glycoproteomics and allied topics. Analytical and Bioanalytical Chemistry.

[174] Campbell MP, Abrahams JL, Rapp E, Struwe WB, Costello CE, Novotny M (2019). The minimum information required for a glycomics experiment (MIRAGE) project: LC guidelines. Glycobiology.

[175] Hanson RL, Hollingsworth MA (2016). Functional Consequences of Differential O-glycosylation of MUC1, MUC4, and MUC16 (Downstream Effects on Signaling). Biomolecules.

[176] Steentoft C, Bennett EP, Clausen H (2013). Glycoengineering of human cell lines using zinc finger nuclease gene targeting: SimpleCells with homogeneous GalNAc O-glycosylation allow isolation of the O-glycoproteome by one-step lectin affinity chromatography. Methods Mol Biol.

[177] Campos D, Freitas D, Gomes J, Magalhaes A, Steentoft C, Gomes C (2015). Probing the O-glycoproteome of gastric cancer cell lines for biomarker discovery. Mol Cell Proteomics.

[178] Yang Z, Halim A, Narimatsu Y, Jitendra Joshi H, Steentoft C, Schjoldager KT (2014). The GalNAc-type O-Glycoproteome of CHO cells characterized by the SimpleCell strategy. Mol Cell Proteomics.

[179] Very N, Lefebvre T, El Yazidi-Belkoura I (2018). Drug resistance related to aberrant glycosylation in colorectal cancer. Oncotarget.

[180] Pinho SS, Oliveira P, Cabral J, Carvalho S, Huntsman D, Gartner F (2012). Loss and recovery of Mgat3 and GnT-III Mediated E-cadherin N-glycosylation is a mechanism involved in epithelial-mesenchymal-epithelial transitions. PLoS One.

[181] Keates AC, Tummala S, Peek RM Jr, Csizmadia E, Kunzli B, Becker K (2008). Helicobacter pylori infection stimulates plasminogen activator inhibitor 1 production by gastric epithelial cells. Infect Immun.

[182] Kim JH, Lee SH, Choi S, Kim U, Yeo IS, Kim SH (2017). Direct analysis of aberrant glycosylation on haptoglobin in patients with gastric cancer. Oncotarget.

[183] Langlois MR, Delanghe JR (1996). Biological and clinical significance of haptoglobin polymorphism in humans. Clin Chem.

[184] Zhang S, Shang S, Li W, Qin X, Liu Y (2016). Insights on N-glycosylation of human haptoglobin and its association with cancers. Glycobiology.

[185] Mann B, Madera M, Klouckova I, Mechref Y, Dobrolecki LE, Hickey RJ (2010). A quantitative investigation of fucosylated serum glycoproteins with application to esophageal adenocarcinoma. Electrophoresis.

[186] Song E, Zhu R, Hammoud ZT, Mechref Y (2014). LC-MS/MS quantitation of esophagus disease blood serum glycoproteins by enrichment with hydrazide chemistry and lectin affinity chromatography. J Proteome Res.

[187] Qiu Y, Patwa TH, Xu L, Shedden K, Misek DE, Tuck M (2008). Plasma glycoprotein profiling for colorectal cancer biomarker identification by lectin glycoarray and lectin blot. J Proteome Res.

[188] Kodera Y, Isobe K, Yamauchi M, Satta T, Hasegawa T, Oikawa S (1993). Expression of carcinoembryonic antigen (CEA) and nonspecific crossreacting antigen (NCA) in gastrointestinal cancer; the correlation with degree of differentiation. Br J Cancer.

[189] Amri R, Bordeianou LG, Sylla P, Berger DL (2013). Preoperative carcinoembryonic antigen as an outcome predictor in colon cancer. J Surg Oncol.

[190] Goldstein MJ, Mitchell EP (2005). Carcinoembryonic antigen in the staging and follow-up of patients with colorectal cancer. Cancer Invest.

[191] Young PE, Womeldorph CM, Johnson EK, Maykel JA, Brucher B, Stojadinovic A (2014). Early detection of colorectal cancer recurrence in patients undergoing surgery with curative intent: current status and challenges. J Cancer.

[192] Locker GY, Hamilton S, Harris J, Jessup JM, Kemeny N, Macdonald JS (2006). ASCO 2006 update of recommendations for the use of tumor markers in gastrointestinal cancer. J Clin Oncol.

[193] Ruibal Morell A (1992). CEA serum levels in non-neoplastic disease. Int J Biol Markers.

[194] Huang C, Zhan T, Liu Y, Li Q, Wu H, Ji D (2015). Glycomic profiling of carcinoembryonic antigen isolated from human tumor tissue. Clin Proteomics.

[195] Chen L, Deng H, Cui H, Fang J, Zuo Z, Deng J (2018). Inflammatory responses and inflammation-associated diseases in organs. Oncotarget.

[196] Arora A, Patil V, Kundu P, Kondaiah P, Hegde AS, Arivazhagan A (2019). Serum biomarkers identification by iTRAQ and verification by MRM: S100A8/S100A9 levels predict tumor-stroma involvement and prognosis in Glioblastoma. Scientific Reports.

[197] Fernandes E, Peixoto A, Neves M, Afonso LP, Santos LL, Ferreira JA (2015). Humoral response against sialyl-Le(a) glycosylated protein species in esophageal cancer: Insights for immunoproteomic studies. Electrophoresis.

[198] Thanki K, Nicholls ME, Gajjar A, Senagore AJ, Qiu S, Szabo C (2017). Consensus Molecular Subtypes of Colorectal Cancer and their Clinical Implications. Int Biol Biomed J.

[199] Cristescu R, Lee J, Nebozhyn M, Kim KM, Ting JC, Wong SS (2015). Molecular analysis of gastric cancer identifies subtypes associated with distinct clinical outcomes. Nat Med.

[200] Koussounadis A, Langdon SP, Um IH, Harrison DJ, Smith VA (2015). Relationship between differentially expressed mRNA and mRNA-protein correlations in a xenograft model system. Scientific Reports.

[201] Sheynkman GM, Shortreed MR, Cesnik AJ, Smith LM (2016). Proteogenomics: Integrating Next-Generation Sequencing and Mass Spectrometry to Characterize Human Proteomic Variation. Annu Rev Anal Chem (Palo Alto Calif).

[202] Wang X, Zhang B (2014). Integrating genomic, transcriptomic, and interactome data to improve Peptide and protein identification in shotgun proteomics. J Proteome Res.

[203] Mun DG, Bhin J, Kim S, Kim H, Jung JH, Jung Y (2019). Proteogenomic Characterization of Human Early-Onset Gastric Cancer. Cancer Cell.

[204] Ferreira JA, Peixoto A, Neves M, Gaiteiro C, Reis CA, Assaraf YG (2016). Mechanisms of cisplatin resistance and targeting of cancer stem cells: Adding glycosylation to the equation. Drug Resist Updat.

